# The biological implications of Yin Yang 1 in the hallmarks of cancer

**DOI:** 10.7150/thno.43481

**Published:** 2020-03-04

**Authors:** Ian Timothy Sembiring Meliala, Rendy Hosea, Vivi Kasim, Shourong Wu

**Affiliations:** 1 The Key Laboratory of Biorheological Science and Technology, Ministry of Education, College of Bioengineering, Chongqing University, Chongqing 400044, China.; 2 The 111 Project Laboratory of Biomechanics and Tissue Repair, College of Bioengineering, Chongqing University, Chongqing 400044, China.; 3 State and Local Joint Engineering Laboratory for Vascular Implants, Chongqing 400044, China.

**Keywords:** Yin Yang 1, hallmarks of cancer, tumorigenesis, transcriptional regulation, post-translational regulation.

## Abstract

Tumorigenesis is a multistep process characterized by the acquisition of genetic and epigenetic alterations. During the course of malignancy development, tumor cells acquire several features that allow them to survive and adapt to the stress-related conditions of the tumor microenvironment. These properties, which are known as hallmarks of cancer, include uncontrolled cell proliferation, metabolic reprogramming, tumor angiogenesis, metastasis, and immune system evasion. Zinc-finger protein Yin Yang 1 (YY1) regulates numerous genes involved in cell death, cell cycle, cellular metabolism, and inflammatory response. YY1 is highly expressed in many cancers, whereby it is associated with cell proliferation, survival, and metabolic reprogramming. Furthermore, recent studies also have demonstrated the important role of YY1-related non-coding RNAs in acquiring cancer-specific characteristics. Therefore, these YY1-related non-coding RNAs are also crucial for YY1-mediated tumorigenesis. Herein, we summarize recent progress with respect to YY1 and its biological implications in the context of hallmarks of cancer.

## Introduction

**Figure 1:**
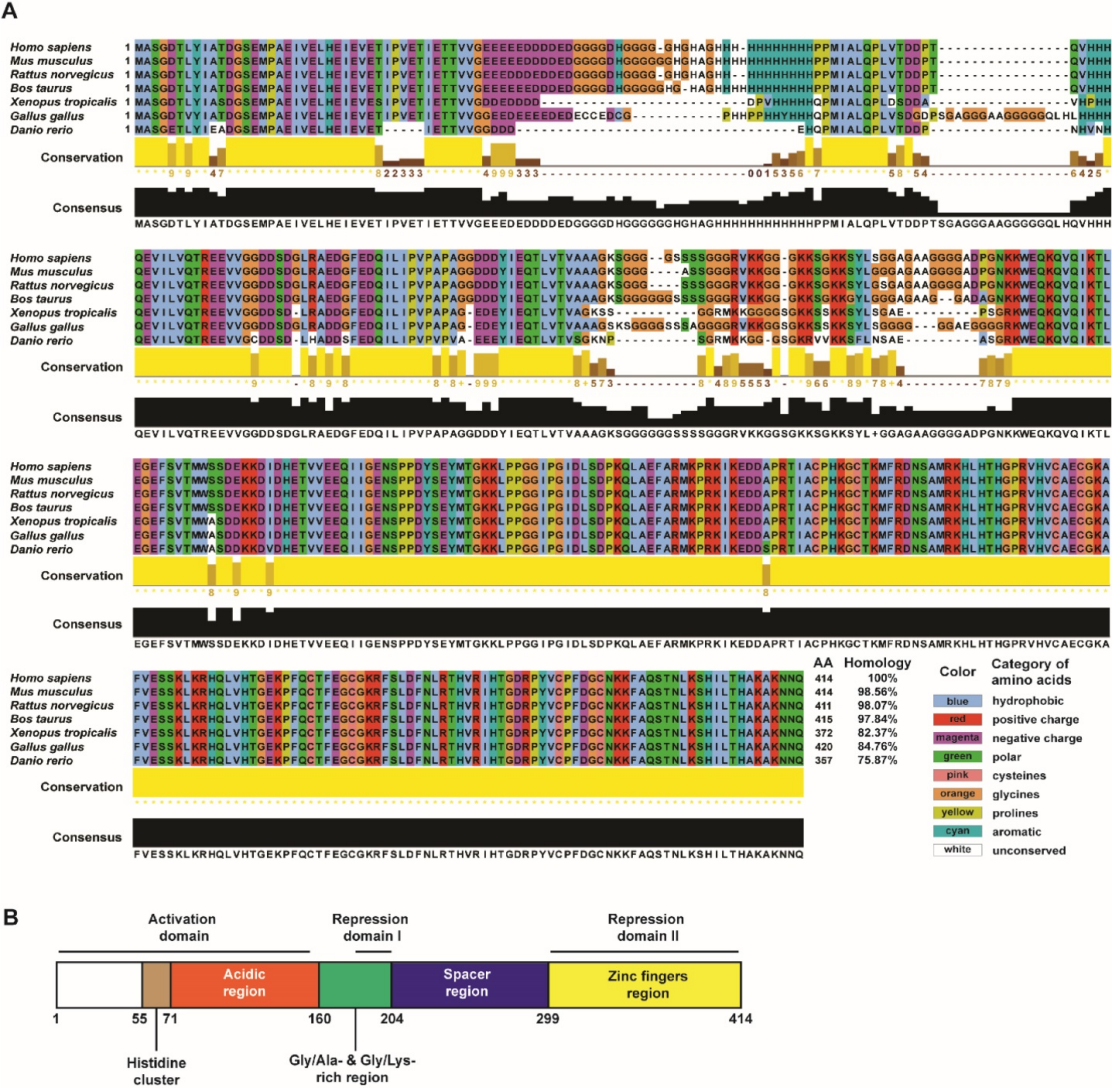
** YY1 structure and conservation among species.** (A) YY1 protein conservation throughout evolutionary development in different vertebrate species. Types of amino acids conserved in all vertebrate species shown are marked with different colors, while unconserved amino acids are shown in white. Blue: hydrophobic amino acids; red: amino acids with positive charge; magenta: amino acids with negative charge; green: polar amino acids; pink: cysteine; orange: glycine; yellow: proline; cyan: aromatic amino acids. (B) Schematic diagram of YY1 structure with its functional domains. The primary amino acid sequences from various species were mined from NCBI Refseq database (NP_033563.2: *Mus musculus*; NP_775412.1: *Rattus norvegicus*; NP_001091550.1: *Bos taurus*; NP_001116880.1: *Xenopus tropicalis;* NP_001026381.1: *Gallus gallus*; NP_997782.1: *Danio rerio*) and sequences were compared to that of human YY1 (NP_003394.1). Multiple-sequence alignments were generated for homologous YY1 protein sequences using UniProt alignment tool (http://www.uniprot.org/align). Jalview was used to visualize and edit the alignment (version 2.11; http://jalview.org).

Yin Yang 1 (YY1) is a zinc-finger protein that belongs to the GLI-Krüppel family. It was first identified from a sequence originally isolated as a repressor of the P5 promoter of adeno-associated virus 1. In humans, YY1 is encoded by the 23-kb YY1 gene located at the chromosomal locus 14q32, which contains five exons and is translated into a 45-kDa protein with 414 amino acids 1-4. *YY1*, which is also known as *NF-E1* in humans and *DELTA* or upstream conserved region-binding protein (*UCRBP*) in mice, is highly conserved in all vertebrates, as well as in some invertebrates (**Figure 1A**) 5.

The name YY1 stands for Yin Yang 1 or positive and negative in ancient Chinese philosophy, and is a reflection of the dual function played by the protein in gene regulation. Depending on the context, YY1 can function as a transcriptional activator or as an inhibitor 1, 6. The dual function of YY1 in regulating gene transcription is due to the presence of an activation domain at the N-terminus, and a repression domain at the C-terminus (**Figure 1B**) 6. YY1 might affect the transcriptional activity of approximately 7% of mammalian genes 7. It traditionally binds to sites with the following consensus: G**CCAT**nTT, Cn**CCAT**nTT, GnCG**ACAT**nTT, CCG**CCAT**nTT, taCG**CCAT**tTTg, CG**CCAT**CTT, CG**CCAT**TTT, and G**CCAT**nTT 8. While 5′-CCAT-3′ and 5′-ACAT-3′ are both conventional YY1 binding sites, YY1 appears to have higher affinity for 5′-CCAT-3′ 9, 10.

YY1-mediated transcriptional repression can occur by direct and competitive binding between YY1 and an activator 11, interference with activator function 12 or by recruitment of a co-repressor involved in chromatin remodeling, such as the Ring1 and YY1 binding protein (RYBP) 13-15. Examples of genes that are regulated by YY1 direct repression are death receptor 5 (*DR5*) 16 and peroxisome proliferator-activated receptor gamma coactivator-1β (*PGC-1β*) 17. Genes that are regulated by co-repression are adenomatous polyposis coli (*APC*) 18 and CXC chemokine receptor type 4 (*CXCR4*) 19. YY1 participates in transcriptional activation also *via* direct promoter binding and interaction with a general transcription factor involved in the formation of a complex with RNA polymerase II 20-22, by masking-unmasking of repression domain 6, or by recruitment of or acting as co-activators 23, 24. Genes that are directly regulated by YY1 include epidermal growth factor receptor (*EGFR*) 25, glucose transporter 3 (*GLUT3*) 21, and glucose-6-phosphate dehydrogenase (*G6PD*) 20. Genes that are regulated by co-activation of YY1 and other transcription factors include erb-b2 receptor tyrosine kinase 2 (*ERBB2*) 26, vascular endothelial growth factor B (*VEGFB*) 27, and p73 28. Besides its role as a transcription factor, YY1 can regulate gene expression at the post-transcriptional stage by controlling the stability and activity of its target genes, including p53 and hypoxia-inducible factor 1-α (HIF-1α) 29, 30. YY1 is also involved in epigenetic regulation of *CDKN2A*, *DTDST*, and *ST6GalNAc6* through chromatin modification by polycomb-group proteins 31, 32, as well as in the post-translational epigenetic modification of histones through recruitment of histone deacetylases (HDACs) 33. Furthermore, it is involved in epigenetic repression of human papilloma virus type 18, whereby it acts as an architectural protein that mediates the physical enhancer-promoter interaction through a chromatin looping-based mechanism 34, 35. The ability of YY1 in forming protein-protein interaction with epigenetic modifiers such as EZH2, p300, and PRMT7 36, is crucial in extending its capacity in regulating gene expression. The direct regulation of YY1 on various genes and its mechanism are shown in **Table 1**.

Considering that YY1 targets a wide range of genes, it is likely to exert critical roles in various biological and physiological functions, including development, proliferation and differentiation, DNA repair and recruitment, epigenetics, genetic imprinting, and oncogenic activity of the cell 37, 38. YY1 mediates translational repression in *Drosophila* embryos during development 39, and is essential for organogenesis of intestinal villi and lung morphogenesis in mouse 40, 41. In genetic imprinting, the difference in sequence motifs allows YY1 to bind both DNA and RNA, and thus act as an adaptor between regulatory RNA and chromatin targets in X-chromosome inactivation 42. It is also involved in neointima formation through regulating p21^WAF/CIP1^-cdk4-Cyclin D1 assembly 43. Furthermore, homozygous deletion of YY1 resulted in embryonic lethality 44; while overexpression of YY1 resulted in severe physiological consequences, such as cardiac hypertrophy and heart failure in transgenic hypertrophic cardiomyopathy (HCM) mice 45, 46.

Tumorigenesis subverts multiple characteristics of normal cells, which enables tumor cells to grow and survive without restraint. Tumor cells not only escape stringent cell cycle regulation—which in turn leads to their unlimited proliferation—but can also acquire resistance against apoptosis. To adapt to their microenvironment, which often lacks oxygen and nutrients supply, tumor cells alter their metabolism, and induce aberrant angiogenesis 47. Critically, tumor cells can alter their characteristics from epithelial to mesenchymal (via epithelial-mesenchymal transition, EMT), and metastasize to other tissues 26. They can also induce inflammation, as well as immune destruction 48. YY1 has been shown to be highly expressed in various tumors. While for some tumors the overexpression of YY1 gives a favorable outcome, elevated YY1 expression is associated primarily with poor prognosis (**Table 2**). Its role in transcriptional as well as post-translational gene regulation is deemed crucial for acquiring tumor cell characteristics (**Table 3**) and, subsequently, for promoting tumorigenesis 36, 49. Moreover, YY1 is also closely related to tumor progression, and its expression in various cancers reflects poor prognosis 36, 50, 51. In this review, we will outline the involvement of YY1 in tumorigenesis, focusing on its role in regulating multiple hallmarks of cancer and the molecular mechanism(s) underlying them.

## YY1 and the hallmarks of cancer

Tumorigenesis is associated with several molecular and biochemical changes that trigger a variety of adaptive mechanisms and the acquisition of several tumor characteristics. These unique traits, which convert a normal cell into a malignant one, are considered the hallmarks of cancer 52, 53. Over the past decade, studies have shed light on the role of YY1 in the acquisition of various cancer hallmarks in developing neoplasms, which will be detailed in the following sections.

### Tumor cell proliferation

A stringent, fine-tuned control over proliferation is essential for maintaining the adequate number of cells in an organism. In contrast, a common feature of cellular transformation and tumor progression is escape from proliferative control. Tumor cells unlimited proliferation is supported by sustained proliferative signaling through oncogene activation, evading growth suppressors, and enabling replicative immortality 53. YY1 controls cell proliferation by activating or repressing specific genes through its transcriptional or post-translational regulatory activity 29, 54. YY1 activates the promoter of the *c-Myc* oncogene and increases its activity in fibroblast cells 55. Moreover, YY1 interacts with protein kinase B (PKB) or AKT and promotes its phosphorylation and conformational change. This event leads to AKT activation by mTOR complex 2 in a phosphoinositide 3-kinase-independent manner and promotes oncogenic signaling 56.

Maintenance of cell homeostasis and proliferation involves interplay between oncogenes and tumor suppressors. The latter exert their function by maintaining cellular growth signal homeostasis, which needs to be disrupted by neoplastic cells. Malignant tumors can overcome the effect of tumor suppressor genes, such as retinoblastoma (Rb) and p53, whose task is to induce cell cycle arrest and apoptosis to avoid uncontrollable cell proliferation or proliferation of cells harboring unrepairable DNA damage 57. YY1 contributes to aberrant control of the cell cycle and apoptosis. YY1 overexpression induces progression into S phase by competitive binding to Rb, a nuclear phosphoprotein, and overcoming Rb-induced cell cycle arrest at the G1/S checkpoint 58. YY1 can negatively regulate p53 expression by facilitating direct binding of p53 to its E3 ligase mouse double minute 2 (MDM2), thereby enhancing p53 ubiquitination and proteasomal degradation, and leading to decreased p53 accumulation 29, 59. YY1 can also positively regulate the p53 homologue p73 by inducing its transcriptional activity in a synergistic manner with E2F transcription factor 1 (E2F1) 28. On the one hand, YY1 decreases the overall transcription activity of p53 by inhibiting the interaction between p53 and its co-activator p300 59. On the other hand, YY1 can maintain p53 transcriptional activity by forming a complex with BRCA2- and CDKN1A-interacting protein (BCCIP). The outcome of p53 transcriptional activity depends on the balance in the YY1/BCCIP/p53 complex, as YY1 inhibits p53RE-mediated p21 transcriptional activation, whereas BCCIP activates it 60.

Cancer stem cells (CSCs) are defined as a subpopulation of cells with high self-renewal capability and pluripotency compared to other subpopulations in tumor tissues. CSCs are highly associated with metastasis, drug resistance, recurrence, as well as poor prognosis, which underlie the difficulty of completely eradicating tumors 61. Proteomic tissue-based datasets have demonstrated an association between YY1 expression and CSC markers (Sox2, Oct4, Bmi1, and Nanog) in different tumors 62. YY1 also directly activates a stem cell marker Krüppel-like factor 4 (KLF4) in non-Hodgkin B-cell lymphoma 63. A specific association of YY1 with different CSC markers has been observed in different types of cancer and could serve as a potential therapeutic approach to suppress the expression of CSCs-related transcription factors.

One strategy used by tumor cells to maintain the proliferative signal is to avoid cellular senescence. *CDKN2A* is one of the most studied tumor suppressor genes associated with cellular senescence. *CDKN2A* encodes p16-INK4a, a cyclin-dependent kinase inhibitor that plays a critical role in cell cycle progression, differentiation, senescence, and apoptosis. YY1 is an epigenetic regulator that forms complexes with HDAC3 and HDAC4. By fine-tuning chromatin modifications, YY1 downregulates *CDKN2A* promoter activity, resulting in cellular escape from senescence 31, 64. In addition to cell cycle regulation, YY1 mediates cellular escape from DNA-damage induced senescence 65 by facilitating DNA repair. YY1 forms a complex with INO80 complex ATPase subunit (INO80), an ATP-dependent chromatin remodeling complex essential in homologous recombination 66. By binding to Holliday junctions, YY1 mediates homologous recombination DNA repair in double-strand breaks 66, which might otherwise lead to DNA damage-induced cell death or senescence.

Together, these studies show the prominent role of YY1 in tumor cell proliferation and reveal the multiple regulatory mechanisms employed by YY1 at transcriptional, post-translational (e.g., protein modification and protein stability), and epigenetic level. Altogether, activation of the proliferative signal, augmented by reduced tumor-suppressive activity, shifts cellular proliferation into overdrive. While reaping the benefits of sustained proliferative signaling requires the cell to be immortal, it is still unclear whether alteration of YY1 alone could give rise to tumor cells with replicative immortality, and thus, further investigation is needed.

### Cell death resistance

Apoptosis is a self-protection mechanism that prevents the proliferation of cells with aberrant gene expression in response to severe, irreparable stress. Evasion from apoptosis, even after DNA damage, genome instability, and oncogene activation, is another important characteristic of malignant transformation. Tumor necrosis factor-related apoptosis-inducing ligand (TRAIL) is a death ligand that triggers apoptosis in a p53-independent manner in various human cancer cell lines 67. YY1 has been shown to mediate the TRAIL-resistant phenotype in a prostate carcinoma cell line by direct binding and inactivation of the promoter of *DR5*, also known as TRAIL receptor 2 (*TRAILR2*), and inhibit its transcription. Inhibition of YY1 increases DR5 expression and in turn sensitizes tumor cells to TRAIL-mediated apoptosis 68. In addition to TRAIL resistance, YY1 induces also resistance to Fas ligand-induced apoptosis by directly repressing the activity of the *FAS* promoter 69. Furthermore, YY1 affects the activity of enhancer of zeste homolog 2 (EZH2) in catalyzing histone H3 lysine 27 trimethylation (H3K27me3) and suppressing tumor suppressor miR-9 activity through DNA methylation. The resulting reduced activity of miR-9 by YY1 causes the activation of nuclear factor-κB (NF-κB), which consequently induces resistance to apoptosis and promotes tumor growth 70. In multiple myeloma cells, YY1 also regulates cell survival by forming complex with RelA and directly repress the promoter of a pro-apoptotic gene, *Bcl2-interacting mediator of cell death (Bim)* 71, and promotes chemoresistance to cytotoxic chemotherapeutic drug bortezomib 72.

Cellular metabolic stress constitutes another type of cell death trigger that regulates survival. Tumor cells utilize a distinct metabolism, characterized by increased aerobic glycolysis and high levels of oxidative stress, to maintain redox homeostasis 73. Though a low to moderate level of reactive oxygen species (ROS) benefits tumor metastasis, high ROS levels are lethal 74. Tumor cells counteract the detrimental effect of oxidative stress by elevating antioxidant defense mechanisms 75. T-cell malignancy 1 (MCT1) stabilizes YY1 mRNA and promotes its activity, which in turn transactivates EGFR expression and inhibits p53 expression. Enhanced EGFR activity leads to increased expression of manganese-dependent superoxide dismutase (MnSOD), a ROS scavenging enzyme, and benefits tumor development by suppressing excessive ROS in tumor cells 76. Furthermore, as p53 serves as the transactivator of the ROS-generating enzymes, quinone oxireductase and proline oxidase, suppression of p53 might lead to further reduction of excessive ROS in tumor cells 77.

Autophagy is another type of cell death that, like apoptosis, involves a self-destructive process. Autophagic cell death is highly regulated. In tumors with a functional apoptotic pathway, autophagy promotes apoptotic tumor suppression in response to metabolic stress. However, in tumors with apoptosis defects, autophagy alleviates metabolic stress and promotes tumor cell survival 78. This happens through catabolic degradation of organelles in response to metabolic stress, and results in sufficient nutrient reserves to sustain tumor cell homeostasis in a nutrient-deprived tumor microenvironment 78. Autophagy has been shown to stimulate apoptosis-induced drug resistance in breast cancer treatment through aurora kinase A (AURKA) inhibition 79, confirming its role in tumorigenesis. During autophagy induction, YY1 co-localizes with transcription factor EB (TFEB) in the nucleus to activate the transcription of autophagy-related genes (*MAP1LC3B*, *Beclin1*, and* Atg5*). YY1 is also known to promote autophagy by stimulating the autophagy regulator SQSTM1 *via* epigenetic downregulation of its inhibitor miR-372 80 or by suppressing miR-30a and dysregulating the NF-κB/SNAIL/YY1/RKIP/PTEN circuit 81, 82. Upregulation of autophagy is known to be an adaptive response in BRAF-inhibitor resistant melanoma. A recent study demonstrated that YY1 knockdown resulted in the sensitization of melanoma cells resistant to vemurafenib, a BRAF-inhibitor 83.

Hence, YY1 aids tumor cells in resisting cell death by tipping the apoptosis-anti-apoptosis balance in favor of cell survival, thereby protecting the cells from damage, and by activating autophagy-mediated survival.

### Deregulation of cellular energetics and metabolic reprogramming

Metabolism is a fundamental physiological process in living cells/organisms that provides them with energy and building blocks. Due to their elevated proliferation rate, tumor cells—especially those located in the center of a solid tumor—often face a severe microenvironment lacking oxygen and nutrients. To satisfy their demand for oxygen and nutrients, tumor cells secrete angiogenic factors to induce angiogenesis. However, the blood vessels formed in this way are usually immature and leaky. This results in a fluctuating and unstable oxygen supply to tumor tissues 84. To meet their demand for large amounts of energy and macromolecules—required for the biosynthesis of organelles necessary to form new cells—tumor cells reprogram their metabolism.

Tumor cell metabolic reprogramming was first observed by Otto Warburg, who noticed that the glucose uptake rate was significantly higher in tumor cells compared to that in normal cells 85. Furthermore, he also found that even in the presence of sufficient oxygen, tumor cells preferred aerobic glycolysis to oxidative phosphorylation in the mitochondria. Recent studies have shown that YY1 is a critical regulator of tumor cell glucose metabolic reprogramming. YY1 induces a metabolic shift by binding directly to the *GLUT3* promoter and activating its transcription 21. This enhances not only the glucose uptake by tumor cells, but also lactate production, which could benefit tumor progression by promoting metastatic potential 86. Furthermore, YY1 might be involved in tumor cell glucose metabolic reprogramming indirectly by promoting HIF-1α stability under hypoxia, which in turn enhances the expression of glucose transporters GLUT1 and GLUT3 87. HIF-1α also transactivates hexokinase-1 and hexokinase-2 in the first step of the glycolytic pathway 88. Furthermore, YY1 enhances the activities of phosphoglycerate kinase 1 (PGK1), the first ATP-generating enzyme in the glycolytic pathway, and pyruvate kinase M2 (PKM2), which catalyzes the final step of glycolysis indirectly through HIF-1α 89. Moreover, YY1 transcriptionally regulates genes associated with mitochondrial energy metabolism, such as those related to the Krebs cycle (*ACO2*, *IDH2*, *OGDH*, *DLD*, and *FH*) and electron transport chain (*ATP5A1*,* ATP5B*, *ATP5F1*, *SDHA*, *SDHB*, *UQCRC2*, *NDUFS1*, *COX2*, *COX5B*, and *COX4l1*) 90. YY1 also indirectly suppresses the Krebs cycle through HIF-1α stabilization by activating pyruvate dehydrogenase kinase 1 (PDK1), which subsequently inactivates pyruvate dehydrogenase (PDH), an important enzyme of the Krebs cycle 91, 92. Finally, YY1 destabilizes p53, which is critical for maintaining the glucose metabolic balance as it activates the p53-induced glycolysis regulator TIGAR 93 and suppresses pyruvate dihydrogenase kinase 2 (PDK2) 94. Hence, YY1 suppresses the level of TIGAR, which results in the increases of the fructose-2,6-bisphosphate level and PFK activity. Increases of PFK activity converts fructose-6-phosphate to fructose-1,6-bisphosphate and thus stimulates glycolysis. Moreover, YY1 positive regulation on the level of PDK2 subsequently suppresses Krebs cycle by inhibiting PDH. Further studies on YY1 regulation of these genes are required to reveal YY1-specific mechanisms that affect mitochondrial energy metabolism.

Anabolic biosynthesis constitutes an inherent and indispensable part of cellular replication, as it is necessary for a cell to replicate all of its contents during mitosis in order to produce two daughter cells. Our previous report showed that YY1 binds to the promoter of *G6PD*, the rate-limiting enzyme in the pentose phosphate pathway (PPP) 20. YY1 promotes *G6PD* transcription and enhances the PPP in tumor cells. This leads to increased production of ribose-5-phosphate, the building block for *de novo* nucleotides biosynthesis, as well as NADPH, an intracellular reducing agent essential for lipid biogenesis. As a result, YY1 enhances PPP promotes tumorigenesis 20. Furthermore, our recent study showed that YY1 could suppress expression of PGC-1β by binding directly to its promoter region. PGC-1β is an activator of medium- and long-chain acyl-CoA dehydrogenases (MCAD and LCAD, respectively), which are crucial for fatty acid β-oxidation. Hence, YY1 can enhance tumor cell lipid accumulation under both hypoxic and normoxic conditions by suppressing β-oxidation of fatty acids 17. A previous study showed that under hypoxic conditions, HIF-1α suppressed PGC-1β expression, and thereby lipid accumulation in tumor cells 95. Considering that YY1 stabilizes HIF-1α under hypoxia, regulation of PGC-1β-mediated fatty acid β-oxidation by YY1 likely occurs in both a direct and HIF-1α-dependent manner. This demonstrates the critical role of YY1 in lipid metabolism in tumor cells regardless of oxygen availability and tumorigenic stage, further supporting the role of YY1 as an important driver of tumorigenesis 17.

**Figure 2:**
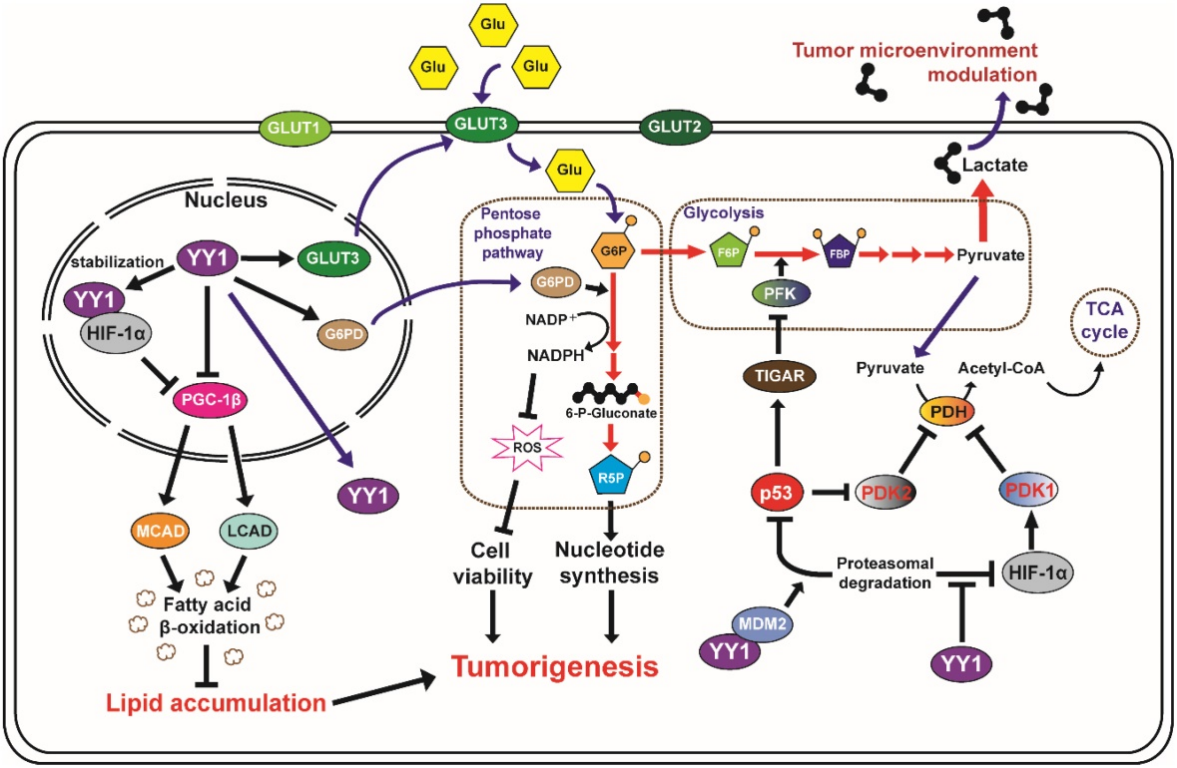
** YY1 and tumor metabolic reprogramming.** YY1 is involved in the tumor metabolic reprogramming, as it regulates glycolysis, glucose uptake and lipid accumulation. YY1 elevates nutrient intake and enhances metabolic activity to meet the demand of biomolecules and maintain energy homeostasis in tumor cells, as well as to promote cell survival via ROS modulation. (Glu: glucose; G6P: glucose 6-phosphate; R5P: ribose 5-phosphate, F6P: fructose 6-phosphate, FBP: fructose 1,6-bisphosphate).

Overall, the role of YY1 in tumor metabolic reprogramming contributes to the adaptability of tumor cells to a changing environment. An altered glucose and lipid catabolism supports tumor cell survival during nutrient-depleted conditions by enabling the utilization of a wider range of carbon substrates (**Figure 2**). At the same time, an altered anabolism supports cell survival during nutrient-replete conditions by accelerating the rate of biosynthesis and reducing the damage incurred by ROS. As excessive intracellular ROS are lethal for tumor cells, various anti-tumor drugs including platinum complexes (cisplatin, carboplatin, and oxaliplatin) and anthracyclines (doxorubicin, epirubicin, and daunorubicin) aim to induce tumor cell death by increasing the intracellular ROS level 74. Therefore, elevated YY1 expression in tumor cells might represent a hurdle during tumor treatment with DNA-damaging drugs. To overcome this problem, a combination treatment with YY1 inhibitor and DNA-damaging anti-tumor drugs might be an option.

### Induction of angiogenesis

Stimulation of angiogenesis is another feature employed by tumor cells to overcome the lack of oxygen and nutrients. Angiogenesis is an intrinsic process stimulated by angiogenesis factors, such as VEGF-A, angiopoietin 1, EGF, fibroblast growth factor 2 (FGF2), platelet-derived growth factor B (PDGFB), and ephrin-B2 (EFNB2) 96. These factors in turn affect the proliferation and migration of endothelial and smooth muscle cells, two major types of cells that form blood vessels, around the primary tumor 97.

The HIF family of transcriptional factors functions as a master gene regulator following exposure to hypoxia. HIF-1α is responsible for the acute hypoxic response. Under normoxic conditions, HIF-1α is hydroxylated at its Pro 402 and Pro 564 residues by prolyl hydroxylase domain (PHD)-containing proteins, utilizing oxygen as the substrate 98. Hydroxylation of HIF-1α results in its ubiquitination and subsequent rapid degradation through the proteasomal pathway. In response to acute hypoxic stimuli, due to lack of oxygen as the substrate, HIF-1α is not hydroxylated, and instead gets accumulated in the nucleus 98, where it forms a heterodimer complex with HIF-1β, whose expression is ubiquitous under both normoxic and hypoxic conditions. The HIF-1 complex, in turn, activates genes promoting angiogenesis, such as *VEGF*, *FGF2*, and *PDGFB*. In addition, HIF-1α can activate genes involved in cell survival and metabolism, such as *PGK*, *CA9*, *BNIP3*, and* GLUT1*, all of which benefit tumor progression 30.

YY1 plays a crucial role in HIF-1α-induced tumor angiogenesis by regulating HIF-1α at post-translational level. Recently, we have shown that YY1 regulates HIF-1α at post-translational level by interacting physically with it and thus suppressing its proteasomal degradation in response to hypoxic conditions 30. Accordingly, YY1 knockdown disrupts the stability of HIF-1α and reduces its accumulation, resulting in the suppression of VEGF and tumor growth factor alpha (TGF-α), and subsequent inhibition of tumor angiogenesis and tumorigenesis. Moreover, YY1 regulation of HIF-1α is independent of the status of p53, which is negatively regulated by YY1 as described above and could inhibit HIF-1α activity by interacting with p300 29, 30. This enables YY1 to induce tumor angiogenesis irrespective of the status of p53, which is frequently mutated and aberrantly downregulated in tumor cells.

Besides regulating the stability of HIF-1α protein, YY1 also serves as a downstream effector of the CXCR4/SDF1 pathway and acts as a direct transcriptional activator of VEGF. Impairment of the CXCR4/SDF1/YY1 pathway alters the expression pattern of VEGF isoforms in osteosarcoma, and results in the downregulation of VEGF-A and upregulation of VEGF-B and VEGF-C. The two latter isoforms cannot activate the angiogenic VEGFR2 receptor, consequently reducing vessel formation and neoangiogenesis 27. YY1 acts also as a co-transcription factor and upregulates the activity of the *VEGF* promoter by forming a complex with HIF-1α that binds to the hormone-response element in the *VEGF* promoter. The end result is increased VEGF expression and enhanced tumor neovascularization 27, 99.

Furthermore, YY1 could induce tumor angiogenesis in a HIF-1-independent manner. Together with the nuclear transcription factor-Y (NFY), YY1 binds to the promoter of *DEK* and activates its transcription. As a key regulator of VEGF expression, DEK upregulation enhances the expression of VEGF, stimulates new blood vessels formation, and promotes angiogenesis *in vivo* 100, 101.

Taken together, existing evidence suggests that YY1 regulation of tumor angiogenesis involves a complex, multi-pathway mechanism based on both direct and indirect activation of angiogenic genes, at the transcriptional as well as post-translational level.

### Activation of invasion and metastasis

Malignant transformation is characterized by local invasion and distant metastases. Transformed cells acquire a motile-invasive phenotype, enabling them to spread via blood or lymph vessels. The induction of EMT is associated with the acquisition of invasive properties and is the first key limiting step in metastasis. Cells undergoing EMT exhibit a characteristic reduced expression of epithelial markers. Of particular importance is the loss of proteins involved in cell-cell contacts such as E-cadherin (*CDH1*). Downregulation of E-cadherin results in detachment from cell-cell junctions, thereby freeing the cells from physical confinement. Unconfined cells are free to invade the underlying basement membrane and enter circulation 102. YY1 is highly expressed in metastatic tumor cells and is regarded as a bona fide inducer of cancer metastasis 103. YY1 mediates the migratory and invasive potential of breast cancer cells by recruiting and interacting with the PRMT7-HDAC3 complex. This results in the reduction of histone trimethylation (H3K4me3) on the *CDH1* promoter and, subsequently, the suppression of E-cadherin expression 104. Moreover, YY1 can induce EMT by regulating twist-related protein 1 (Twist1), a key activator of EMT. YY1 binds directly to the upstream promoter sequence of heterogeneous nuclear ribonucleoprotein M (*hnRNPM*), an RNA-binding protein that participates in the processing of pre-mRNAs into mature RNAs, and inhibits its promoter activity. This, in turn, leads to the release of Twist1 from the suppressive effect of hnRNPM 105. YY1 also induces the expression of snail through direct binding on its 3′ enhancer region 106. Furthermore, YY1 enhances the expression of vimentin, plausibly through direct binding with its promoter 106. Both snail and vimentin are crucial EMT markers, thus YY1 regulation on them further convince close relation between YY1 and EMT.

Although EMT enables potentially invasive cancer cells to spread from the site of the primary tumor by entering the circulatory system, most of these cells will not survive. Only a small proportion manages to succeed in establishing metastasis in distant organs. Subcutaneous xenograft experiments with lung cancer and gastric cancer cell lines showed that *YY1* overexpression enhanced lung metastatic potential, and resulted in a lower survival rate 30, 51. Intravenous injection of YY1-knocked-down A549 cells resulted in decreased tumor cells colonization in lung compared to control cells 30.

The link between YY1 and metastasis has been further confirmed by clinical samples from melanoma and gastric cancer patients. Examination of samples from melanoma patient revealed that, compared to primary melanoma, the level of YY1 mRNA was higher in metastatic melanomas 107. Immunohistochemistry on samples from gastric cancer patients showed that YY1 expression correlated positively with lymph node metastasis, distant metastasis, and advanced tumor-node-metastasis 51. Collectively, this evidence supports the notion that elevated YY1 expression contributes to the acquisition of an EMT phenotype and tumor metastasis.

### Genome instability and mutation

In the course of tumorigenesis, neoplastic lesions develop into malignant tumors accompanied by the accumulation of genomic mutations. The types of genomic alterations driving tumorigenesis vary in size from single point mutations to whole-chromosome gains and losses. Cancer genomes are generally dynamic owing to genome instability. Genome instability can be defined as the tendency of tumor cells to acquire mutations as a result of defective DNA maintenance. Failure to repair extensive DNA damage can result in point mutations that activate an oncogene or inactivate a tumor suppressor gene, and hence acts as a driving force towards malignancy. Occasionally, further mutations involving genes required to maintain chromosome stability will result in a phenotype known as chromosomal instability, which favors the generation of additional mutations in oncogenes or tumor suppressor genes 108. Notably, a constantly changing genome fuels intercellular heterogeneity and is a major driving force of tumor evolution and adaptation to challenges arising from the tumor microenvironment 109. Intercellular heterogeneity within a tumor, resulting from genomic instability, provides abundant starting material for clonal selection, and a strong selective pressure eventually favors clones carrying factors essential for cell survival.

Although YY1 is not directly known to prompt the initiation of genomic instability, YY1 is a remarkable predictor of the outcome of breast cancer clonal selection, particularly in estrogen receptor (ER)-positive breast cancer patients undergoing hormone therapy. Through co-localization with ERα, YY1 assists the binding of ERα to the enhancer region, a specific active regulatory region associated with H3K27ac marks, on downstream genes and contributes to their transcription. In the background of hormone therapy aimed at inhibiting estrogen production in breast cancer, YY1 contributes to clonal selection by maintaining the binding of ERα-YY1 to the enhancer of SLC9A3R1, a hormone therapy-resistant gene. Thus, YY1 expression leads to the survival of tumor cells even under strong selective pressure, such as long-term estrogen deprivation by aromatase inhibitors, suggesting a role of YY1 in non-responder patients 23. Aside from a report by Wu et al. that demonstrated the need for the YY1/INO80 complex to prevent the formation of polyploidy and chromatid aberrations 66, little is known about the role of YY1 in genome instability.

### Tumor-promoting inflammation and immunosuppression

Chronic tissue inflammation in cancer is defined as an aberrantly prolonged form of protective tissue repair. Immune cells infiltrate the site of injury, mounting a lasting immune response to combat the intruding pathogen or molecule. In contrast to the anti-tumor activity of acute inflammation, chronic inflammation is a well-recognized tumor-promoting condition. Prolonged inflammation enables most of the core cellular and molecular adaptations required for tumorigenesis such as the release of DNA-damaging ROS, thus augmenting the risk of malignant transformation. Innate inflammatory immune cells recruited by tumor cells are found in the ROS-enriched microenvironment, where they secrete soluble growth factors and chemoattractants 48. In contrast to a balanced inflammatory response that recruits leukocytes to eliminate foreign cells by ROS-mediated bacterial killing in the inflamed area 110, the unresolved inflammation cascade in tumor cells leads to a switch from tumor immunosurveillance to tumor-promoting inflammation, tumor initiation, and cancer progression 111. Hence, instead of killing tumor cells, tumor-related inflammation promotes tumorigenesis.

YY1 was reported to interact with RelB and p50 in glioblastoma cells. This interaction “turns on” the RelB-dependent chronic inflammation switch, which then results in upregulation of proinflammatory cytokines IL1β, IL6, IL8, and oncostatin M. The proinflammatory signaling induced by these mediators enhances cell proliferation, invasiveness, tumor angiogenesis, as well as resistance to drugs and apoptosis. In addition, continuous secretion of these cytokines leads to the recruitment and activation of glioma-associated macrophages, a marker of immunosuppressive microenvironment resistant to immunotherapy 112. Though it is unknown whether YY1 activates NF-κB, a major transcription factor for inflammatory gene expression, the reverse (i.e. regulation of YY1 by NF-κB) is known to occur. Activation of NF-κB stimulates YY1 transcription by direct binding of p65 to the NF-κB binding site on the *YY1* promoter 113.

Chronic inflammation also allows tumors to recruit stromal cells and to form a microenvironment niche, which aids tumor cells in eventually escaping the immune system. This, in turn, makes it difficult to specifically identify and eliminate tumor cells by recognizing the expression of tumor-specific antigens before tumor cells can cause harm 114. The cancer immune microenvironment is constituted of various stromal innate immune cells, including neutrophils, macrophages, mast cells, dendritic cells, and natural killer cells. Among them, a unique subset called tumor-associated macrophages (TAMs) is recognized for its characteristic plasticity, which enables the TAMs to acquire a number of distinct phenotypes and contribute in different ways to various stages of tumorigenesis. TAMs are characterized by a molecular signature reminiscent of alternatively activated M2 macrophages with immunosuppressive phenotype 115. YY1 has been reported to downregulate miR-125a, thereby causing a shift from classically activated M1 macrophages to M2 macrophages. This impairs the immune system and its ability to prevent tumorigenesis 116. YY1 can bind to the cyclooxygenase *COX2* promoter following macrophage activation. Overexpression of YY1 in macrophages increases the production of COX2, an activator of the pro-tumoral activity of macrophages, resulting in polarization towards M2 macrophages and in the ability of the tumor to evade the immune surveillance machinery 117, 118.

Adaptive immune response relies on the ability of cytotoxic T-cells to recognize foreign or mutated antigens displayed on transformed cells and to subsequently induce T-cell-mediated cell death. Tumor cells can evade immune destruction by either direct or indirect attenuation of the killing activity of infiltrating immune cells. Tumor cells directly create an immunosuppressive environment by secreting TGF-β and other immunosuppressive factors, such as IL10, HGF, IDO, and PGE2 77. Furthermore, YY1 might positively regulate the expression of programmed death ligand 1 (PD-L1), whose expression is correlated with poor clinical response to cell-mediated anti-tumor therapy, through crosstalk between YY1 and PD-L1 signaling pathways 119-121. As a negative regulator of p53, YY1 might be able to suppress the expression of miR-34a, a downstream target of p53. The decrease of miR-34a in turn increases PD-L1 expression, as it could suppress PD-L1 expression by binding to its 3′ untranslated region (3′-UTR) 119. YY1 could also directly activate *c-Myc*, and *IL6*, a positive regulator of signal transducer and activator of transcription 3 (STAT3), as well as stabilize HIF-1α protein, these factors subsequently increase *PD-L1* transcription 120. Collectively, YY1 is involved in ROS-mediated DNA mutagenesis, the generation of an immunosuppressive microenvironment, as well as the debilitation of the capability of the immune system to recognize nascent transformed cells. These findings indicate the possibility of combining cancer immunotherapy with YY1 inhibition to sensitize cancer cells to immunotherapeutic agents.

## YY1-related non-coding RNAs (ncRNAs) and the hallmarks of cancer

Accumulating evidence demonstrates that a large portion of the human genome is transcribed into ncRNAs, such as microRNAs (miRNAs), long non-coding RNAs (lncRNAs), and circular RNAs (circRNAs), which play a crucial role in gene regulation at both transcriptional and post-transcriptional stages 122, 123. With only 19-22 bp, miRNAs are important post-transcriptional regulators of gene expression that act *via* complementary base pairing to their target sites within the 3′-UTR of mRNAs. Depending on their complementarity, binding of miRNAs to mRNAs can induce either translational repression or mRNA degradation 124, 125. Growing evidence points to reciprocal regulation between YY1 and miRNAs. On the one hand, YY1 is a negative regulator of let-7a, a tumor suppressor miRNA that inhibits the expression of anti-apoptotic protein B-cell lymphoma-extra-large (BCL-xL) in chemoresistance in acute myeloid leukemia 126; YY1 is also a negative regulator of miR-29b, a suppressor of tumor proliferation in rhabdomyosarcoma 125. On the other hand, miR-7 and miR-181 act as tumor suppressors by binding directly to the 3′-UTR of YY1 mRNA, thus inhibiting its translation and preventing YY1 protein accumulation 127, 128. Known miRNAs regulated by YY1 and miRNAs that regulate YY1 are listed in **Table 4** and **Table 5**, respectively.

As a large class of transcripts with <200 bp, lncRNAs indirectly modulate gene expression at the post-transcriptional level by functioning as a sponge of specific miRNAs. They can also interact with transcription factors or epigenetic proteins to regulate target gene expression, or promote mRNA translation of a specific target gene. Increasing evidence suggests the involvement of lncRNAs in a variety of biological processes, including cell proliferation, tumorigenesis, and invasiveness 129. A recent report showed that the lncRNA nasopharyngeal carcinoma copy number amplified transcript-1 (NPCCAT1), whose expression increased in nasopharyngeal carcinoma, bound directly to the 5′-UTR of YY1 mRNA, causing the upregulation of YY1 translation and subsequently increased cell growth and migration 130.

Recently discovered, circRNAs are highly stable, circular (3′ and 5′ terminal joining) ncRNAs that regulate miRNAs. A circRNA binds to miRNAs, prevents their function, and terminates the suppression of target mRNAs 131, 132. Dysregulated circRNAs have been identified in some types of cancers, suggesting the important role of circRNAs in tumorigenesis and in regulating hallmarks of cancer 133. During EMT, the production of hundreds of circRNAs, including SMARCA5, POLE2, SHPRH, SMAD2, and ATXN2, is dynamically regulated by the alternative splicing factor quaking (QKI). QKI exerts pleiotropic effects, such as increased cell migration, invasion, and EMT 134. Remarkably, YY1 binds to the super-enhancer and promoter of QKI and, together with the p65/p300 complex, activates the transcription of *QKI*. Thus, the fact that YY1 can regulate a pleiotropic factor such as QKI implies that the scale of YY1-mediated regulation of circRNAs and miRNAs might exceed beyond what is presently known 135.

## Conclusion

**Figure 3:**
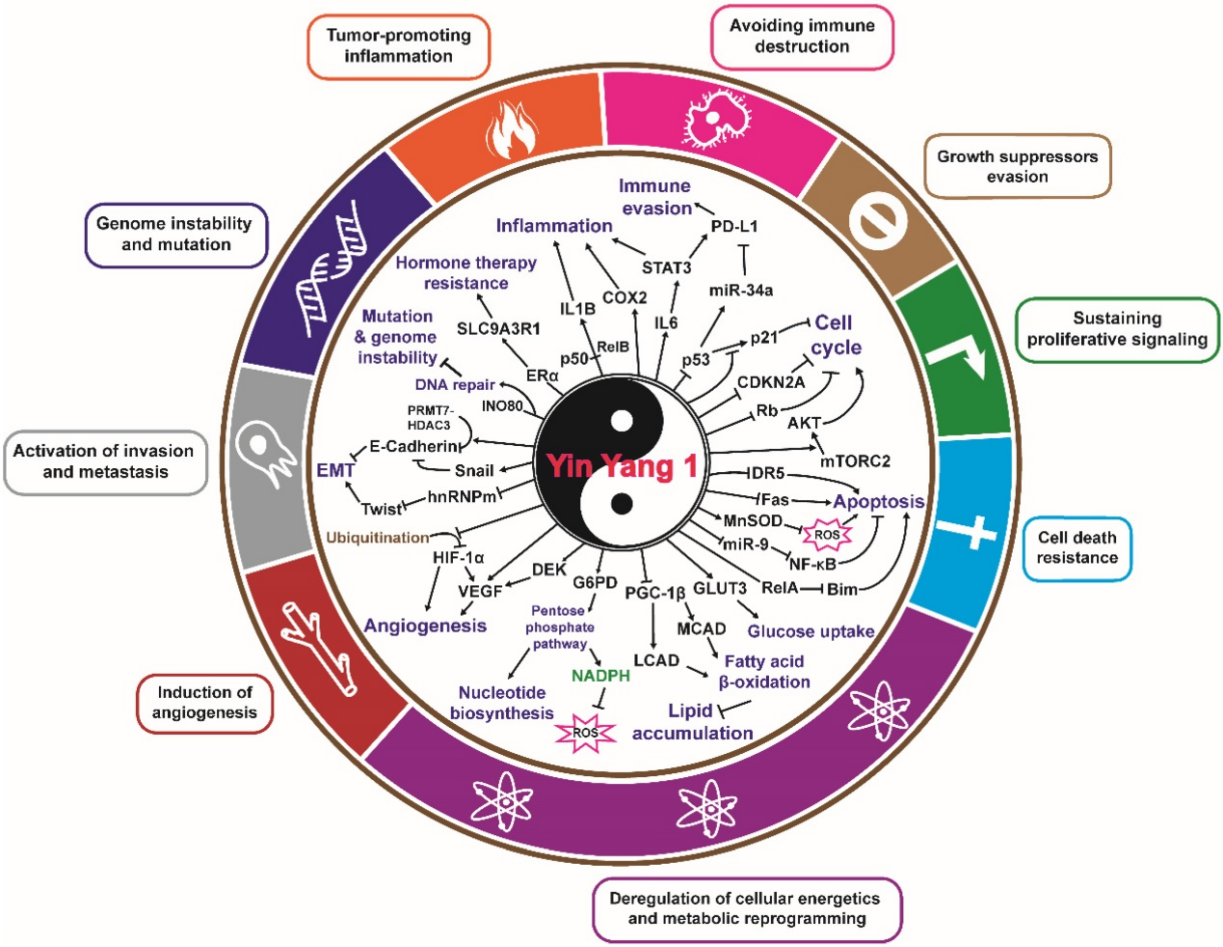
** YY1 and the hallmarks of cancer.** Direct and indirect roles of YY1 in the hallmarks of cancer. YY1 is involved in tumorigenesis by regulating distinct hallmarks of cancer: growth suppressors evasion, sustaining proliferative signaling, cell death resistance, deregulation of cellular energetics and metabolic reprogramming, induction of angiogenesis, activation of invasion and metastasis, genome instability and mutation, tumor promoting inflammation, and avoiding immune destruction.

Since its discovery, YY1 has emerged as an important factor in regulating the expression of a large number of genes, including ncRNAs. The studies discussed in this review emphasize the role of YY1 as a crucial regulator of target genes that influence the 'hallmarks of cancer' 52, 53. YY1 both activates and suppresses the expression of a number of oncogenes and tumor suppressors involved in various cellular functions, including proliferation, redox homeostasis, DNA damage response, apoptosis, angiogenesis, metastasis, and immunosuppression (**Figure 3**). Considering that YY1 overexpression is observed in many cancers and has various biological implications with respect to the hallmarks of cancer, it is an attractive approach to utilize YY1 as a novel target for therapeutic interventions. However, although most evidence supports oncogenic function of YY1, there are few reports about opposing roles of YY1 in tumorigenesis 49. Furthermore, as shown in **Table 2**, while YY1 upregulation majorly related with poor prognosis, some cases showed the opposite. The mechanisms underlying this paradox, as well as whether it is related with the dual function of YY1 in regulating gene expression, remains to be solved. It is also crucial to reveal whether the role of YY1 as an oncogene or tumor suppressor is related with the cancer types, or even subtypes of cancers, especially when designing anti-tumor therapeutic strategy. Clearly, defining the molecular mechanism underlying the contrasting behavior of YY1 in cancer remains an interesting task.

Even in the absence of ongoing clinical trials with YY1 direct inhibitors, several YY1-inhibiting agents are under investigation for their potential use as anti-cancer therapies, either alone or in combination with other drugs. Previous studies showed that nitric oxide donors such as diethylenetriamine NONOate (DETA-NONOate) are encouraging for the development of YY1 inhibitors that can overcome immune and chemotherapy resistance 106, 136-140. Several anti-tumor therapeutic agents that have been used clinically, including immunotherapeutic agent such as rituximab 16 and genotoxic agents such as cisplatin, etoposide, vincristine 68, can suppress YY1 expression; however, their molecular mechanism in regulating YY1, especially whether they can directly inhibit YY1, remains unclear. Furthermore, previous studies reported the tumor suppressive role of YY1 in some cancers 54, 141. In some pancreatic cancers, YY1 could inhibit tumor proliferation, angiogenesis and metastasis by directly repressing tubulin polymerization-promoting protein (*TPPP*) and cyclin-dependent kinase inhibitor 3 *(CDKN3)* promoter activity 11, 141, 142, and high expression of YY1 is related with better prognosis 143. These facts should also be considered for determining the suitability of YY1-targeted therapy.

Another important issue to consider is the presence of Yin Yang 2 (*YY2*), which has only been discovered recently 144. *YY2* is a retroposed copy duplicated from YY1 mRNA located on chromosome X, and therefore is highly homologous to *YY1* in terms of nucleotide and amino acid sequences (65% DNA sequence identity and 56% amino acid identity). As it had been retroposed from YY1 mRNA during the divergence of placental mammals from other vertebrates, *YY2* does not possess any introns in its mRNA sequence 144, 145. YY2 was recently reported to have a function distinct from that of YY1, and has been classified as an intrinsically disordered protein 146-148. Due to the similarity with YY1 at the level of the zinc finger region, YY2 generally interacts with the same molecular partners as YY1 148. However, previous studies on YY2 have revealed an antagonist function to YY1 in regulating p53 activity and cell proliferation 146, 149, 150. Unlike YY1, YY2 expression is downregulated in tumor tissues, and might act as a tumor suppressor. The distinct function of YY2 could arise from a difference in the N-terminal domain 148. Therefore, due to the high homology between YY1 and YY2, in the basic research, attention should be paid to the specificity of probes and antibodies used to detect them; while the specificity of any YY1 inhibitor is a crucial issue that needs to be to be considered when designing drugs for cancer therapy. This stresses the need for a better understanding of YY1/YY2 dynamics to shed light on the distinct roles of YY1 and YY2, as well as the interaction between them and the possibility of their competitive binding in the genomic DNA binding sites 151. Therefore, novel tools for dissecting the dynamics of YY1/YY2 should be developed and used to direct the targeting of YY1, eventually leading to a significant addition to currently available cancer treatment regimens.

Overall, the fact that a single protein, YY1, regulates several hallmarks of cancer indicates the relevant role of YY1 in tumorigenesis. Furthermore, while there are still several crucial concerns need to be solved before its clinical application, these facts also emphasize the prognostic and therapeutic potential of YY1 for anti-tumor therapy.

## Figures and Tables

**Table 1:** Genes directly regulated by YY1.

Gene	Regulation	Mechanism	Ref
*AKT*	Upregulated	Protein stabilization	56
*APC*	Downregulated	Epigenetic repression	18
*Atg5*	Upregulated	Recruitment of / being co-activator	83
*Beclin1*	Upregulated	Recruitment of / being co-activator	83
*Bim*	Downregulated	Recruitment of / being co-repressor	71
*CDKN2A*	Downregulated	Epigenetic repression	31, 64
*CDKN3*	Downregulated	Direct repression on promoter region	11
*c-Myc*	Upregulated	Direct activation on promoter region	55
*COX2*	Upregulated	Direct activation on promoter region	117, 118
*CXCR4*	Downregulated	Recruitment of / being co-activator	19
*DEK*	Upregulated	Direct activation on promoter region	100, 101
*DR5*	Downregulated	Direct repression on promoter region	16
*DTDST*	Downregulated	Epigenetic repression	31, 32
*CDH1*	Downregulated	Epigenetic repression	104
*EGFR*	Upregulated	Direct activation on promoter region	25
*ERBB2*	Upregulated	Recruitment of / being co-activator	26
*Fas*	Downregulated	Direct repression on promoter region	69
*G6PD*	Upregulated	Direct activation on promoter region	20
*GLUT3*	Upregulated	Direct activation on promoter region	21
*HIF-1α*	Upregulated	Protein stabilization	30
*hnRNPM*	Downregulated	Direct activation on promoter region	105
*HPV18*	Downregulated	Epigenetic repression	34, 35
*IL6*	Upregulated	Direct activation on promoter region	120
*KLF4*	Upregulated	Direct activation on promoter region	63
*MAP1LC3B*	Upregulated	Recruitment of / being co-activator	83
*miR-125a*	Downregulated	Recruitment of / being co-repressor	116
*miR-30a*	Downregulated	Direct repression on promoter region	81, 82
*miR-372*	Downregulated	Epigenetic repression	80
*miR-9*	Downregulated	Epigenetic repression	70
*p21*	Downregulated	Direct activation on promoter region	60
*p53*	Downregulated	Protein stabilization, interference with activator function	29,59
*p73*	Upregulated	Recruitment of / being co-activator	28
*PGC-1β*	Downregulated	Direct repression on promoter region	17
*RelB*	Upregulated	Recruitment of / being co-activator	112
*Rb*	Downregulated	Protein stabilization	58
*RYBP*	Downregulated	Recruitment of / being co-repressor	13-15
*ST6GalNAc6*	Downregulated	Epigenetic repression	31, 32
*TPPP*	Downregulated	Direct repression on promoter region	142
*VEGF*	Upregulated	Recruitment of / being co-activator	27, 99
*VEGFB*	Upregulated	Direct activation on promoter region	27

**Abbreviations:** APC: adenomatous polyposis coli; Atg5: autophagy related 5; Bim: Bcl2-interacting mediator of cell death; CDH1: Cadherin-1; CDKN2A: cyclin-dependent kinase inhibitor 2A; CDKN3: cyclin-dependent kinase inhibitor 3; COX2: cyclooxygenase 2; CXCR4: chemokine receptor type 4; DR5: death receptor 5; DTDST: diastrophic dysplasia sulfate transporter; EGFR: epidermal growth factor receptor; ERBB2: erb-b2 receptor tyrosine kinase 2; G6PD: glucose-6-phosphate dehydrogenase; GLUT3: glucose transporter 3; HIF-1α: hypoxia-inducible factor 1-α; hnRNPM: heterogeneous nuclear ribonucleoprotein M; HPV18: human papilloma virus strains 18; IL6: interleukin 6; KLF4: Kruppel-like factor 4; MAP1LC3B: microtubule associated proteins 1A/1B light chain 3B; PGC-1β: peroxisome proliferator-activated receptor gamma coactivator-1β; Rb: Retinoblastoma; RYBP: RING1 and YY1 binding protein; ST6GalNAc6: ST6 N-acetylgalactosaminide alpha-2,6-sialyltransferase 6; TPPP: tubulin polymerization-promoting protein; VEGF: vascular endothelial growth factor; VEGFB: vascular endothelial growth factor B.

**Table 2:** YY1 expression in various cancers.

Cancer types	YY1 expression level	Prognosis	Ref
Bladder	Upregulated	n/a	152
Breast	Downregulated	n/a	153
	Upregulated	Good	154
Cervical	Upregulated	n/a	155
Colon	Upregulated	n/a	156
	Upregulated	Poor	127
Esophageal	Upregulated	Poor	157
Gastric	Upregulated	Poor	51, 158
Glioma	Upregulated	n/a	159
Hodgkin lymphoma	Upregulated	n/a	160
Leukemia	Upregulated	n/a	161
	Upregulated	Poor	162
	Upregulated	n/a	163
Liver	Upregulated	Poor	164
Lung	Upregulated	n/a	163
Melanoma	Upregulated	n/a	107
Multiple myeloma	Upregulated	Poor	72
Nasopharynx	Downregulated	Good	165
Non-Hodgkin lymphoma	Upregulated	n/a	166
	Upregulated	Poor	167
	Downregulated	Good	168
Osteosarcoma	Upregulated	Poor	50
Ovarian	Upregulated	n/a	169
Pancreatic	Upregulated	Good	143
	Upregulated	Poor	170
Prostate	Upregulated	Good	170
Renal	Upregulated	Poor	171
Sarcoma	Upregulated	n/a	172
	Upregulated	Poor	50
Testicular seminoma	Upregulated	n/a	173
Thyroid	Upregulated	n/a	174

**Abbreviations:** n/a: not available

**Table 3:** Biological implications of YY1 on hallmarks of cancer.

Related Genes	Implications on hallmarks of cancer	Effect	Mechanism	Ref
*CDKN2A*	Tumor cell proliferation	Cellular escape from senescence	Downregulates CDKN2A promoter activity	31
*c-Myc*	Tumor cell proliferation	Increased cell proliferation	Activates the promoter of *c-Myc*	55
*AKT*	Tumor cell proliferation	Increased cell proliferation	Promotes AKT phosphorylation and conformational change	56
*p21*	Tumor cell proliferation	Increased cell proliferation	YY1/BCCIP complex regulates p53RE-mediated transcription of p21	60
*Rb*	Tumor cell proliferation	Reduces Rb-induced cell cycle arrest at G_1_/S checkpoint	Direct competitive binding with Rb	58
*p53*	Tumor cell proliferation	Increased cell proliferation	Induces proteasomal degradation of p53	29, 59
*DR5*	Resisting cell death	Resistance to TRAIL-induced apoptosis	Negatively regulates DR5 transcription	68
*FAS*	Resisting cell death	Resistance to Fas-induced apoptosis	Represses *FAS* expression by binding to its silencer region	69, 175
*NF-κB*	Resisting cell death	Increased cell proliferation	YY1-mediated epigenetic silencing of miR-9 leading to increased NF-κB expression	70
*Bim*	Resisting cell death	Reduced apoptosis	YY1 forms complex with RelA and directly represses the activity of *Bim* promoter.	71
*MCT1*	Resisting cell death, metabolic reprogramming	Tumor cell survival under oxidative stress	YY1 is targeted by MCT1 and mediates EGFR/MnSOD (activation) and p53 (inhibition) gene expression	76
*GLUT3*	Metabolic reprogramming	Enhances the aerobic glucose metabolism in tumor cells	Activates GLUT3 transcription by direct binding on its promoter in p53-independent manner	21
*G6PD*	Metabolic reprogramming	Enhances the PPP pathway	Activates G6PD transcription by direct binding on its promoter in p53-independent manner	20
*VEGF*	Inducing angiogenesis	Promote neoangiogenesis	Forming complex with HIF-1α and activates VEGF expression	27
*HIF-1α*	Inducing angiogenesis	Expression of HIF-1α target genes and promotion of neoangiogenesis	YY1 has physical interaction with HIF-1α and regulates its expression in p53-independent manner.	30
*MMP14*	Activating invasion and metastasis	Promotes metastasis	Promotes MMP14 transcription through direct binding on its promoter	51
*CDH1*	Activating invasion and metastasis	Decreased cell-cell junction, induction of EMT	Represses E-cadherin expression by reducing histone trimethylation (H3K4me3) level through recruitment of PRMT7-HDAC3 complex	104
*INO80*	Genome instability and mutation	Genome stabilization	YY1 form complex with INO80 to regulate HDR in tumor cell	66
*IL1B*	Tumor-promoting inflammation	Induction of inflammatory response	Interacts with RelB and p50, activating the transcription of IL1 proinflammatory cytokines	112
*PD-L1*	Evading immune destruction	Resistance to killing by immune cells	Regulates the expression of PD-L1 through various pathways	120

**Abbreviations:** Bim: Bcl2-interacting mediator of cell death; CDH1: Cadherin-1; CDKN2A: cyclin-dependent kinase Inhibitor 2A; DR5: death receptor 5; G6PD: glucose-6-phosphate dehydrogenase; GLUT3: glucose transporter 3; HDR: homologous recombination DNA repair; HIF-1α: hypoxia-inducible factor 1 alpha; IL1B: interleukin 1β; INO80: INO80 complex ATPase subunit; MCT1: T-cell malignancy 1; MMP14: matrix metalloproteinase-14; NF-kB: nuclear factor-kB; PD-L1: programmed death ligand 1; Rb: retinoblastoma; VEGF: vascular endothelial growth factor.

**Table 4:** Non-coding RNAs regulated by YY1.

Gene/ promoter	Role in tumorigenesis	YY1 regulatory activity	Reported malignancy	Hallmarks	Ref
Let-7a	Tumor-suppressor	Repression	AML	Cell death resistance	126
miR-1260b	Oncogene	Activation	NSCLC	Tumor cell proliferation, cell death resistance	176
miR-135b	Oncogene	Activation	Pancreatic	Tumor cell proliferation, cell death resistance	170
miR-140	Tumor-suppressor	Activation	Breast	Tumor cell proliferation, cell death resistance	177
miR-141	Oncogene	Repression	Nasopharyngeal	Tumor cell proliferation, cell death resistance	165
miR-29b	Tumor-suppressor	Repression	Rhabdomyo-sarcoma	Tumor cell proliferation	125
miR-320a	Tumor-suppressor	Activation	Colorectal	Tumor cell proliferation, cell death resistance	178
miR-361	Tumor-suppressor	Repression	Endometrial	Tumor cell proliferation, activation of invasion & metastasis	179
miR-489	Tumor-suppressor	Repression	Pancreatic	Activation of invasion & metastasis	180
miR-500a-5p	Tumor-suppressor	Repression	Colorectal	Tumor cell proliferation, cell death resistance, invasion & metastasis	181
miR-520c-3p	Tumor-suppressor	Activation	Lung	Tumor cell proliferation, cell death resistance	182
miR-526b-3p	Tumor-suppressor	Repression	Colorectal	Tumor cell proliferation	183
miR-5590-3p	Oncogene	Repression	Breast	Tumor cell proliferation, cell death resistance, invasion & metastasis	184
miR-9	Tumor-suppressor	Repression	Melanoma	Tumor cell proliferation, activation of invasion & metastasis	107
DDX11-AS1	Oncogene	Activation	Colorectal	Tumor cell proliferation, cell death resistance, invasion & metastasis	185
LINC00673	Oncogene	Activation	Breast	Tumor cell proliferation, cell death resistance	186
lncRNA-ARAP1-AS1	Oncogene	Activation	Colorectal	Invasion & metastasis	187
lncRNA-PVT1	Oncogene	Activation	Lung	Tumor cell proliferation	188
lncRNA-SOX2OT	Tumor-suppressor	Repression	Pancreatic	Tumor cell proliferation	189
lncRNA-TUG1	Oncogene	Activation	Glioma	Tumor cell proliferation, cell death resistance	190
lncMER52A	Oncogene	Activation	Liver	Activation of invasion & metastasis	191
lncMCM3AP-AS1	Oncogene	Activation	Lung	Tumor cell proliferation, activation of invasion & metastasis, induction of angiogenesis	192
Multiple circRNAs	Oncogene and/or tumor-suppressor	Activation	Liver	Tumor cell proliferation, invasion & metastasis	135

**Abbreviations:** AML: acute myeloid leukemia; ARAP1-AS1: ArfGAP with RhoGAP domain, ankyrin repeat and PH domain 1-antisense RNA 1; DDX11-AS1: DDX11-antisense RNA 1; Let-7a: lethal-7 a; LINC00673: long intergenic non-protein coding RNA 673; lncRNA: long non-coding RNA; MCM3AP-AS1: mini chromosome maintenance complex component 3 associated protein, antisense RNA 1; miR: microRNA; NSCLC: non-small-cell lung carcinoma; PVT1: plasmacytoma variant translocation 1; QKI: quaking; SOX2OT: SRY-Box 2 overlapping transcript; TUG1: taurine up-regulated 1.

**Table 5:** Non-coding RNAs as YY1 regulators.

Gene/promoter	Role in tumorigenesis	Effect on YY1 activity	Reported malignancy	Hallmarks	Ref
miR-101	Tumor-suppressor	Repression	Gastric	Tumor cell proliferation	193
miR-141-3p	Tumor-suppressor	Repression	Thyroid	Tumor cell proliferation, cell death resistance, invasion & metastasis	194
miR-181	Tumor-suppressor	Repression	Cervical	Tumor cell proliferation, cell death resistance	128
miR-186	Tumor-suppressor	Repression	Glioblastoma, Lung	Cell death resistance, tumor cell proliferation, invasion & metastasis	195 196
miR-193a-5p	Tumor-suppressor	Repression	Endometrial	Tumor cell proliferation, invasion & metastasis	18
miR-215	Tumor-suppressor	Repression	Colorectal	Tumor cell proliferation, invasion & metastasis	197
miR-218	Tumor-suppressor	Repression	Glioma	Tumor cell proliferation, invasion & metastasis	198
miR-29a	Tumor-suppressor	Repression	Lung	Tumor cell proliferation, cell death resistance, invasion & metastasis	199
miR-34a	Tumor-suppressor	Repression	Neuroblastoma, Esophageal, Gastric	Tumor cell proliferation, cell death resistance, invasion & metastasis	200 201 202
miR-381	Tumor-suppressor	Repression	Ovarian	Tumor cell proliferation, invasion & metastasis	203
miR-544	Tumor-suppressor	Repression	Thyroid	Tumor cell proliferation, invasion & metastasis	204
miR-5590-3p	Tumor-suppressor	Repression	Breast	Tumor cell proliferation, cell death resistance, invasion & metastasis	184
miR-635	Tumor-suppressor	Repression	NSCLC	Invasion & metastasis	205
miR-7	Tumor-suppressor	Repression	Colorectal	Tumor cell proliferation, cell death resistance	127
miR-7-5p	Tumor-suppressor	Repression	Glioblastoma	Cell death resistance	206
lncRNA-CASC15	Oncogene	Activation	AML	Tumor cell proliferation, cell death resistance	207
lncRNA-NPCCAT1	Oncogene	Activation	Nasopharyngeal	Tumor cell proliferation	130
circ-CTNNB1	Oncogene	Activation	Gastric, prostate, colorectal	Tumor cell proliferation, invasion & metastasis	208
circ-HIPK3	Oncogene	Activation	Colorectal	Tumor cell proliferation, cell death resistance, invasion & metastasis	209

**Abbreviations:** CASC15: cancer susceptibility candidate 15; circ: circular RNA; CTNNB1: catenin beta 1; HIPK3: homeodomain interacting protein kinase 3; lncRNA: long non-coding RNA; miR: micro RNA; NPCCAT1: nasopharyngeal carcinoma copy number amplified transcript-1; NSCLC: non-small-cell lung carcinoma.

## Abbreviations

APC: adenomatous polyposis coli
AURKA: aurora kinase A
BCL-xL: B-cell lymphoma-extra-large
Bim: Bcl2-interacting mediator of cell death
BCCIP: BRCA2- and CDKN1A-interacting protein
CDH1: Cadherin-1
CDKN2A: cyclin-dependent kinase inhibitor 2A
CDKN3: cyclin-dependent kinase inhibitor 3
circRNA: circular RNA
COX: cyclooxygenase
CSC: cancer stem cell
CXCR4: CXC chemokine receptor type 4
DR5: death receptor 5
DTDST: diastrophic dysplasia sulfate transporter
E2F1: E2F transcription factor 1
EGFR: epidermal growth factor receptor
EMT: epithelial-mesenchymal transition
EFNB2: ephrin-B2
ER: estrogen receptor
ERBB2: erb-b2 receptor tyrosine kinase 2
EZH2: enhancer of zeste homolog 2
FGF2: fibroblast growth factor 2
G6PD: glucose-6-phosphate dehydrogenase
GLUT: glucose transporter
HDAC: histone diacetylase
HIF-1α: hypoxia-inducible factor 1-α
KLF4: Kruppel-like factor 4
LCAD: long-chain acyl-CoA dehydrogenase
lncRNA: long non-coding RNA
MCAD: medium-chain acyl-CoA dehydrogenase
MCT1: T-cell malignancy 1
ΜDΜ2: mouse double minute 2
miRNA: micro RNA
MnSOD: manganese-dependent superoxide dismutase
ncRNA: non-coding RNA
NF-κB: nuclear factor-κB
NFY: nuclear transcription factor-Y
NPCCAT1: nasopharyngeal carcinoma copy number amplified transcript-1
PD-L1: programmed death ligand 1
PDGFB: platelet-derived growth factor B
PDH: pyruvate dehydrogenase
PDK1: pyruvate dehydrogenase kinase 1
PDK2: pyruvate dehydrogenase kinase 2
PFK: phosphofructokinase
PGC-1β: peroxisome proliferator-activated receptor gamma coactivator-1β
PGK1: phosphoglycerate kinase 1
PHD: prolyl hydroxylase domain
PKM2: pyruvate kinase M2
PPP: pentose phosphate pathway
PRMT7: protein arginine methyltransferase 7
QKI: quaking
Rb: retinoblastoma
ROS: reactive oxygen species
SDF1: stromal cell-derived factor 1
ST6GalNAc6: N-acetylgalactosamine α 2,6 sialyltransferase isoenzyme 6
STAT3: signal transducer and activator of transcription 3
TAM: tumor-associated macrophage
TFEB: transcription factor EB
TGF: tumor growth factor
TPPP: tubulin polymerization-promoting protein
TRAIL: tumor necrosis factor-related apoptosis-inducing ligand
Twist1: twist-related protein 1
UCRBP: upstream conserved region-binding protein
VEGF: vascular endothelial growth factor
YY1: Yin Yang 1
YY2: Yin Yang 2

## References

[1] Shi Y, Seto E, Chang LS, Shenk T (1991). Transcriptional repression by YY1, a human GLI-Kruppel-related protein, and relief of repression by adenovirus E1A protein. Cell.

[2] Zhu W, Lossie AC, Camper SA, Gumucio DL (1994). Chromosomal localization of the transcription factor YY1 in the mouse and human. Mamm Genome.

[3] Yao YL, Dupont BR, Ghosh S, Fang Y, Leach RJ, Seto E (1998). Cloning, chromosomal localization and promoter analysis of the human transcription factor YY1. Nucleic Acids Res.

[4] Gorecki A, Bonarek P, Gorka AK, Figiel M, Wilamowski M, Dziedzicka-Wasylewska M (2015). Intrinsic disorder of human Yin Yang 1 protein. Proteins.

[5] Gordon S, Akopyan G, Garban H, Bonavida B (2006). Transcription factor YY1: structure, function, and therapeutic implications in cancer biology. Oncogene.

[6] Lee JS, See RH, Galvin KM, Wang J, Shi Y (1995). Functional interactions between YY1 and adenovirus E1A. Nucleic Acids Res.

[7] Atchison M, Basu A, Zaprazna K, Papasani M (2011). Mechanisms of Yin Yang 1 in oncogenesis: the importance of indirect effects. Crit Rev Oncog.

[8] Figiel M, Gorecki A (2017). Physical Interaction of Human Yin Yang 1 Protein with DNA. Crit Rev Oncog.

[9] Yant SR, Zhu W, Millinoff D, Slightom JL, Goodman M, Gumucio DL (1995). High affinity YY1 binding motifs: identification of two core types (ACAT and CCAT) and distribution of potential binding sites within the human beta globin cluster. Nucleic Acids Res.

[10] Hyde-DeRuyscher RP, Jennings E, Shenk T (1995). DNA binding sites for the transcriptional activator/repressor YY1. Nucleic Acids Res.

[11] Liu D, Zhang J, Wu Y, Shi G, Yuan H, Lu Z (2018). YY1 suppresses proliferation and migration of pancreatic ductal adenocarcinoma by regulating the CDKN3/MdM2/P53/P21 signaling pathway. Int J Cancer.

[12] Xu J, De Zhu J, Ni M, Wan F, Gu JR (2002). The ATF/CREB site is the key element for transcription of the human RNA methyltransferase like 1(RNMTL1) gene, a newly discovered 17p13.3 gene. Cell Res.

[13] Wilkinson FH, Park K, Atchison ML (2006). Polycomb recruitment to DNA *in vivo* by the YY1 REPO domain. Proc Natl Acad Sci U S A.

[14] Bajusz I, Henry S, Sutus E, Kovacs G, Pirity MK (2019). Evolving Role of RING1 and YY1 Binding Protein in the Regulation of Germ-Cell-Specific Transcription. Genes (Basel).

[15] Zhan S, Wang T, Ge W, Li J (2018). Multiple roles of Ring 1 and YY1 binding protein in physiology and disease. J Cell Mol Med.

[16] Bonavida B (2007). Rituximab-induced inhibition of antiapoptotic cell survival pathways: implications in chemo/immunoresistance, rituximab unresponsiveness, prognostic and novel therapeutic interventions. Oncogene.

[17] Li Y, Kasim V, Yan X, Li L, Meliala ITS, Huang C (2019). Yin Yang 1 facilitates hepatocellular carcinoma cell lipid metabolism and tumor progression by inhibiting PGC-1beta-induced fatty acid oxidation. Theranostics.

[18] Yang Y, Zhou L, Lu L, Wang L, Li X, Jiang P (2013). A novel miR-193a-5p-YY1-APC regulatory axis in human endometrioid endometrial adenocarcinoma. Oncogene.

[19] Lee BC, Lee TH, Zagozdzon R, Avraham S, Usheva A, Avraham HK (2005). Carboxyl-terminal Src kinase homologous kinase negatively regulates the chemokine receptor CXCR4 through YY1 and impairs CXCR4/CXCL12 (SDF-1alpha)-mediated breast cancer cell migration. Cancer Res.

[20] Wu S, Wang H, Li Y, Xie Y, Huang C, Zhao H (2018). Transcription Factor YY1 Promotes Cell Proliferation by Directly Activating the Pentose Phosphate Pathway. Cancer Res.

[21] Wang Y, Wu S, Huang C, Li Y, Zhao H, Kasim V (2018). Yin Yang 1 promotes the Warburg effect and tumorigenesis via glucose transporter GLUT3. Cancer Sci.

[22] Usheva A, Shenk T (1996). YY1 transcriptional initiator: protein interactions and association with a DNA site containing unpaired strands. Proc Natl Acad Sci U S A.

[23] Patten DK, Corleone G, Gyorffy B, Perone Y, Slaven N, Barozzi I (2018). Enhancer mapping uncovers phenotypic heterogeneity and evolution in patients with luminal breast cancer. Nat Med.

[24] Deng Z, Cao P, Wan MM, Sui G (2010). Yin Yang 1: a multifaceted protein beyond a transcription factor. Transcription.

[25] Yin D, Ogawa S, Kawamata N, Leiter A, Ham M, Li D (2013). miR-34a functions as a tumor suppressor modulating EGFR in glioblastoma multiforme. Oncogene.

[26] Allouche A, Nolens G, Tancredi A, Delacroix L, Mardaga J, Fridman V (2008). The combined immunodetection of AP-2alpha and YY1 transcription factors is associated with ERBB2 gene overexpression in primary breast tumors. Breast Cancer Res.

[27] de Nigris F, Crudele V, Giovane A, Casamassimi A, Giordano A, Garban HJ (2010). CXCR4/YY1 inhibition impairs VEGF network and angiogenesis during malignancy. Proc Natl Acad Sci U S A.

[28] Wu S, Murai S, Kataoka K, Miyagishi M (2008). Yin Yang 1 induces transcriptional activity of p73 through cooperation with E2F1. Biochem Biophys Res Commun.

[29] Sui G, Affar el B, Shi Y, Brignone C, Wall NR, Yin P (2004). Yin Yang 1 is a negative regulator of p53. Cell.

[30] Wu S, Kasim V, Kano MR, Tanaka S, Ohba S, Miura Y (2013). Transcription factor YY1 contributes to tumor growth by stabilizing hypoxia factor HIF-1alpha in a p53-independent manner. Cancer Res.

[31] Wang X, Feng Y, Xu L, Chen Y, Zhang Y, Su D (2008). YY1 restrained cell senescence through repressing the transcription of p16. Biochim Biophys Acta.

[32] Huang HC, Chao CC, Wu PH, Chung HY, Lee HY, Suen CS (2019). Epigenetic silencing of the synthesis of immunosuppressive Siglec ligand glycans by NF-kappaB/EZH2/YY1 axis in early-stage colon cancers. Biochim Biophys Acta Gene Regul Mech.

[33] Thomas MJ, Seto E (1999). Unlocking the mechanisms of transcription factor YY1: are chromatin modifying enzymes the key?. Gene.

[34] Pentland I, Campos-Leon K, Cotic M, Davies KJ, Wood CD, Groves IJ (2018). Disruption of CTCF-YY1-dependent looping of the human papillomavirus genome activates differentiation-induced viral oncogene transcription. PLoS Biol.

[35] Agrawal P, Heimbruch KE, Rao S (2018). Genome-Wide Maps of Transcription Regulatory Elements and Transcription Enhancers in Development and Disease. Compr Physiol.

[36] Shi J, Hao A, Zhang Q, Sui G (2015). The role of YY1 in oncogenesis and its potential as a drug target in cancer therapies. Curr Cancer Drug Targets.

[37] Nicholson S, Whitehouse H, Naidoo K, Byers RJ (2011). Yin Yang 1 in human cancer. Crit Rev Oncog.

[38] Weintraub AS, Li CH, Zamudio AV, Sigova AA, Hannett NM, Day DS (2017). YY1 Is a Structural Regulator of Enhancer-Promoter Loops. Cell.

[39] Atchison L, Ghias A, Wilkinson F, Bonini N, Atchison ML (2003). Transcription factor YY1 functions as a PcG protein *in vivo*. EMBO J.

[40] Boucherat O, Landry-Truchon K, Berube-Simard FA, Houde N, Beuret L, Lezmi G (2015). Epithelial inactivation of Yy1 abrogates lung branching morphogenesis. Development.

[41] Kumar N, Srivillibhuthur M, Joshi S, Walton KD, Zhou A, Faller WJ (2016). A YY1-dependent increase in aerobic metabolism is indispensable for intestinal organogenesis. Development.

[42] Jeon Y, Lee JT (2011). YY1 tethers Xist RNA to the inactive X nucleation center. Cell.

[43] Santiago FS, Ishii H, Shafi S, Khurana R, Kanellakis P, Bhindi R (2007). Yin Yang-1 inhibits vascular smooth muscle cell growth and intimal thickening by repressing p21WAF1/Cip1 transcription and p21WAF1/Cip1-Cdk4-cyclin D1 assembly. Circ Res.

[44] Donohoe ME, Zhang X, McGinnis L, Biggers J, Li E, Shi Y (1999). Targeted disruption of mouse Yin Yang 1 transcription factor results in peri-implantation lethality. Mol Cell Biol.

[45] Stauffer BL, Dockstader K, Russell G, Hijmans J, Walker L, Cecil M (2015). Transgenic over-expression of YY1 induces pathologic cardiac hypertrophy in a sex-specific manner. Biochem Biophys Res Commun.

[46] Sucharov CC, Mariner P, Long C, Bristow M, Leinwand L (2003). Yin Yang 1 is increased in human heart failure and represses the activity of the human alpha-myosin heavy chain promoter. J Biol Chem.

[47] Nakazawa MS, Keith B, Simon MC (2016). Oxygen availability and metabolic adaptations. Nat Rev Cancer.

[48] Elinav E, Nowarski R, Thaiss CA, Hu B, Jin C, Flavell RA (2013). Inflammation-induced cancer: crosstalk between tumours, immune cells and microorganisms. Nat Rev Cancer.

[49] Zhang Q, Stovall DB, Inoue K, Sui G (2011). The oncogenic role of Yin Yang 1. Crit Rev Oncog.

[50] de Nigris F, Zanella L, Cacciatore F, De Chiara A, Fazioli F, Chiappetta G (2011). YY1 overexpression is associated with poor prognosis and metastasis-free survival in patients suffering osteosarcoma. BMC Cancer.

[51] Zheng L, Chen Y, Ye L, Jiao W, Song H, Mei H (2017). miRNA-584-3p inhibits gastric cancer progression by repressing Yin Yang 1- facilitated MMP-14 expression. Sci Rep.

[52] Hanahan D, Weinberg RA (2000). The hallmarks of cancer. Cell.

[53] Hanahan D, Weinberg RA (2011). Hallmarks of cancer: the next generation. Cell.

[54] Khachigian LM (2018). The Yin and Yang of YY1 in tumor growth and suppression. Int J Cancer.

[55] Riggs KJ, Saleque S, Wong KK, Merrell KT, Lee JS, Shi Y (1993). Yin-yang 1 activates the c-myc promoter. Mol Cell Biol.

[56] Zhang Q, Wan M, Shi J, Horita DA, Miller LD, Kute TE (2016). Yin Yang 1 promotes mTORC2-mediated AKT phosphorylation. J Mol Cell Biol.

[57] Sherr CJ, McCormick F (2002). The RB and p53 pathways in cancer. Cancer Cell.

[58] Petkova V, Romanowski MJ, Sulijoadikusumo I, Rohne D, Kang P, Shenk T (2001). Interaction between YY1 and the retinoblastoma protein. Regulation of cell cycle progression in differentiated cells. J Biol Chem.

[59] Gronroos E, Terentiev AA, Punga T, Ericsson J (2004). YY1 inhibits the activation of the p53 tumor suppressor in response to genotoxic stress. Proc Natl Acad Sci U S A.

[60] Sui Y, Wu T, Li F, Wang F, Cai Y, Jin J (2019). YY1/BCCIP Coordinately Regulates P53-Responsive Element (p53RE)-Mediated Transactivation of p21(Waf1/Cip1). Int J Mol Sci.

[61] Lobo NA, Shimono Y, Qian D, Clarke MF (2007). The biology of cancer stem cells. Annu Rev Cell Dev Biol.

[62] Kaufhold S, Garban H, Bonavida B (2016). Yin Yang 1 is associated with cancer stem cell transcription factors (SOX2, OCT4, BMI1) and clinical implication. J Exp Clin Cancer Res.

[63] Morales-Martinez M, Valencia-Hipolito A, Vega GG, Neri N, Nambo MJ, Alvarado I (2019). Regulation of Kruppel-Like Factor 4 (KLF4) expression through the transcription factor Yin-Yang 1 (YY1) in non-Hodgkin B-cell lymphoma. Oncotarget.

[64] Feng Y, Wang X, Xu L, Pan H, Zhu S, Liang Q (2009). The transcription factor ZBP-89 suppresses p16 expression through a histone modification mechanism to affect cell senescence. FEBS J.

[65] Van Nguyen T, Puebla-Osorio N, Pang H, Dujka ME, Zhu C (2007). DNA damage-induced cellular senescence is sufficient to suppress tumorigenesis: a mouse model. J Exp Med.

[66] Wu S, Shi Y, Mulligan P, Gay F, Landry J, Liu H (2007). A YY1-INO80 complex regulates genomic stability through homologous recombination-based repair. Nat Struct Mol Biol.

[67] Shankar S, Srivastava RK (2004). Enhancement of therapeutic potential of TRAIL by cancer chemotherapy and irradiation: mechanisms and clinical implications. Drug Resist Updat.

[68] Baritaki S, Huerta-Yepez S, Sakai T, Spandidos DA, Bonavida B (2007). Chemotherapeutic drugs sensitize cancer cells to TRAIL-mediated apoptosis: up-regulation of DR5 and inhibition of Yin Yang 1. Mol Cancer Ther.

[69] Pothoulakis C, Torre-Rojas M, Duran-Padilla MA, Gevorkian J, Zoras O, Chrysos E (2018). CRHR2/Ucn2 signaling is a novel regulator of miR-7/YY1/Fas circuitry contributing to reversal of colorectal cancer cell resistance to Fas-mediated apoptosis. Int J Cancer.

[70] Tsang DP, Wu WK, Kang W, Lee YY, Wu F, Yu Z (2016). Yin Yang 1-mediated epigenetic silencing of tumour-suppressive microRNAs activates nuclear factor-kappaB in hepatocellular carcinoma. J Pathol.

[71] Potluri V, Noothi SK, Vallabhapurapu SD, Yoon SO, Driscoll JJ, Lawrie CH (2013). Transcriptional repression of Bim by a novel YY1-RelA complex is essential for the survival and growth of Multiple Myeloma. PLoS One.

[72] Huerta-Yepez S, Liu H, Baritaki S, Del Lourdes Cebrera-Munoz M, Rivera-Pazos C, Maldonado-Valenzuela A (2014). Overexpression of Yin Yang 1 in bone marrow-derived human multiple myeloma and its clinical significance. Int J Oncol.

[73] Cairns RA, Harris IS, Mak TW (2011). Regulation of cancer cell metabolism. Nat Rev Cancer.

[74] Gorrini C, Harris IS, Mak TW (2013). Modulation of oxidative stress as an anticancer strategy. Nat Rev Drug Discov.

[75] Diehn M, Cho RW, Lobo NA, Kalisky T, Dorie MJ, Kulp AN (2009). Association of reactive oxygen species levels and radioresistance in cancer stem cells. Nature.

[76] Tseng HY, Chen YA, Jen J, Shen PC, Chen LM, Lin TD (2017). Oncogenic MCT-1 activation promotes YY1-EGFR-MnSOD signaling and tumor progression. Oncogenesis.

[77] Turley SJ, Cremasco V, Astarita JL (2015). Immunological hallmarks of stromal cells in the tumour microenvironment. Nat Rev Immunol.

[78] Karantza-Wadsworth V, Patel S, Kravchuk O, Chen G, Mathew R, Jin S (2007). Autophagy mitigates metabolic stress and genome damage in mammary tumorigenesis. Genes Dev.

[79] Mani SA, Guo W, Liao MJ, Eaton EN, Ayyanan A, Zhou AY (2008). The epithelial-mesenchymal transition generates cells with properties of stem cells. Cell.

[80] Feng L, Ma Y, Sun J, Shen Q, Liu L, Lu H (2014). YY1-MIR372-SQSTM1 regulatory axis in autophagy. Autophagy.

[81] Yang C, Zhang JJ, Peng YP, Zhu Y, Yin LD, Wei JS (2017). A Yin-Yang 1/miR-30a regulatory circuit modulates autophagy in pancreatic cancer cells. J Transl Med.

[82] Bonavida B (2018). Linking Autophagy and the Dysregulated NFkappaB/ SNAIL/YY1/RKIP/PTEN Loop in Cancer: Therapeutic Implications. Crit Rev Oncog.

[83] Du J, Ren W, Yao F, Wang H, Zhang K, Luo M (2019). YY1 cooperates with TFEB to regulate autophagy and lysosomal biogenesis in melanoma. Mol Carcinog.

[84] Jain RK (2005). Normalization of tumor vasculature: an emerging concept in antiangiogenic therapy. Science.

[85] Vander Heiden MG, Cantley LC, Thompson CB (2009). Understanding the Warburg effect: the metabolic requirements of cell proliferation. Science.

[86] Hirschhaeuser F, Sattler UG, Mueller-Klieser W (2011). Lactate: a metabolic key player in cancer. Cancer Res.

[87] Macheda ML, Rogers S, Best JD (2005). Molecular and cellular regulation of glucose transporter (GLUT) proteins in cancer. J Cell Physiol.

[88] Patra KC, Wang Q, Bhaskar PT, Miller L, Wang Z, Wheaton W (2013). Hexokinase 2 is required for tumor initiation and maintenance and its systemic deletion is therapeutic in mouse models of cancer. Cancer Cell.

[89] Semenza GL (2010). HIF-1: upstream and downstream of cancer metabolism. Curr Opin Genet Dev.

[90] Park A, Lee J, Mun S, Kim DJ, Cha BH, Moon KT (2017). Identification of Transcription Factor YY1 as a Regulator of a Prostate Cancer-Specific Pathway Using Proteomic Analysis. J Cancer.

[91] Kim JW, Tchernyshyov I, Semenza GL, Dang CV (2006). HIF-1-mediated expression of pyruvate dehydrogenase kinase: a metabolic switch required for cellular adaptation to hypoxia. Cell Metab.

[92] Papandreou I, Cairns RA, Fontana L, Lim AL, Denko NC (2006). HIF-1 mediates adaptation to hypoxia by actively downregulating mitochondrial oxygen consumption. Cell Metab.

[93] Bensaad K, Tsuruta A, Selak MA, Vidal MN, Nakano K, Bartrons R (2006). TIGAR, a p53-inducible regulator of glycolysis and apoptosis. Cell.

[94] Liu J, Zhang C, Hu W, Feng Z (2015). Tumor suppressor p53 and its mutants in cancer metabolism. Cancer Lett.

[95] Huang Li T, Li X Zhang L, Sun L He X (2014). HIF-1-mediated suppression of acyl-CoA dehydrogenases and fatty acid oxidation is critical for cancer progression. Cell Rep.

[96] Semenza GL (2003). Angiogenesis in ischemic and neoplastic disorders. Annu Rev Med.

[97] Weis SM, Cheresh DA (2011). Tumor angiogenesis: molecular pathways and therapeutic targets. Nat Med.

[98] Lee JW, Bae SH, Jeong JW, Kim SH, Kim KW (2004). Hypoxia-inducible factor (HIF-1)alpha: its protein stability and biological functions. Exp Mol Med.

[99] Yang W, Li Z, Qin R, Wang X, An H, Wang Y (2019). YY1 Promotes Endothelial Cell-Dependent Tumor Angiogenesis in Hepatocellular Carcinoma by Transcriptionally Activating VEGFA. Front Oncol.

[100] Zhang Y, Liu J, Wang S, Luo X, Li Y, Lv Z (2016). The DEK oncogene activates VEGF expression and promotes tumor angiogenesis and growth in HIF-1alpha-dependent and -independent manners. Oncotarget.

[101] Sitwala KV, Adams K, Markovitz DM (2002). YY1 and NF-Y binding sites regulate the transcriptional activity of the dek and dek-can promoter. Oncogene.

[102] Dongre A, Weinberg RA (2019). New insights into the mechanisms of epithelial-mesenchymal transition and implications for cancer. Nat Rev Mol Cell Biol.

[103] Wang W, Li D, Sui G (2017). YY1 Is an Inducer of Cancer Metastasis. Crit Rev Oncog.

[104] Yao R, Jiang H, Ma Y, Wang L, Wang L, Du J (2014). PRMT7 induces epithelial-to-mesenchymal transition and promotes metastasis in breast cancer. Cancer Res.

[105] Yang T, An Z, Zhang C, Wang Z, Wang X, Liu Y (2019). hnRNPM, a potential mediator of YY1 in promoting the epithelial-mesenchymal transition of prostate cancer cells. Prostate.

[106] Cho AA, Bonavida B (2017). Targeting the Overexpressed YY1 in Cancer Inhibits EMT and Metastasis. Crit Rev Oncog.

[107] Zhao G, Li Q, Wang A, Jiao J (2015). YY1 regulates melanoma tumorigenesis through a miR-9 ~ RYBP axis. J Exp Clin Cancer Res.

[108] Bastians H (2015). Causes of Chromosomal Instability. Recent Results Cancer Res.

[109] Lee JK, Choi YL, Kwon M, Park PJ (2016). Mechanisms and Consequences of Cancer Genome Instability: Lessons from Genome Sequencing Studies. Annu Rev Pathol.

[110] Coussens LM, Zitvogel L, Palucka AK (2013). Neutralizing tumor-promoting chronic inflammation: a magic bullet?. Science.

[111] Pesic M, Greten FR (2016). Inflammation and cancer: tissue regeneration gone awry. Curr Opin Cell Biol.

[112] Waters MR, Gupta AS, Mockenhaupt K, Brown LN, Biswas DD, Kordula T (2019). RelB acts as a molecular switch driving chronic inflammation in glioblastoma multiforme. Oncogenesis.

[113] Wang H, Hertlein E, Bakkar N, Sun H, Acharyya S, Wang J (2007). NF-kappaB regulation of YY1 inhibits skeletal myogenesis through transcriptional silencing of myofibrillar genes. Mol Cell Biol.

[114] Swann JB, Smyth MJ (2007). Immune surveillance of tumors. J Clin Invest.

[115] Arlauckas SP, Garren SB, Garris CS, Kohler RH, Oh J, Pittet MJ (2018). Arg1 expression defines immunosuppressive subsets of tumor-associated macrophages. Theranostics.

[116] Zhao JL, Huang F, He F, Gao CC, Liang SQ, Ma PF (2016). Forced Activation of Notch in Macrophages Represses Tumor Growth by Upregulating miR-125a and Disabling Tumor-Associated Macrophages. Cancer Res.

[117] Joo M, Wright JG, Hu NN, Sadikot RT, Park GY, Blackwell TS (2007). Yin Yang 1 enhances cyclooxygenase-2 gene expression in macrophages. Am J Physiol Lung Cell Mol Physiol.

[118] Nakanishi Y, Nakatsuji M, Seno H, Ishizu S, Akitake-Kawano R, Kanda K (2011). COX-2 inhibition alters the phenotype of tumor-associated macrophages from M2 to M1 in ApcMin/+ mouse polyps. Carcinogenesis.

[119] Cortez MA, Ivan C, Valdecanas D, Wang X, Peltier HJ, Ye Y (2016). PDL1 Regulation by p53 via miR-34. J Natl Cancer Inst.

[120] Hays E, Bonavida B (2019). YY1 regulates cancer cell immune resistance by modulating PD-L1 expression. Drug Resist Updat.

[121] Ricklefs FL, Alayo Q, Krenzlin H, Mahmoud AB, Speranza MC, Nakashima H (2018). Immune evasion mediated by PD-L1 on glioblastoma-derived extracellular vesicles. Sci Adv.

[122] Derrien T, Johnson R, Bussotti G, Tanzer A, Djebali S, Tilgner H (2012). The GENCODE v7 catalog of human long noncoding RNAs: analysis of their gene structure, evolution, and expression. Genome Res.

[123] Long Y, Wang X, Youmans DT, Cech TR (2017). How do lncRNAs regulate transcription?. Sci Adv.

[124] Bracken CP, Scott HS, Goodall GJ (2016). A network-biology perspective of microRNA function and dysfunction in cancer. Nat Rev Genet.

[125] Wang H, Garzon R, Sun H, Ladner KJ, Singh R, Dahlman J (2008). NF-kappaB-YY1-miR-29 regulatory circuitry in skeletal myogenesis and rhabdomyosarcoma. Cancer Cell.

[126] Chen Y, Jacamo R, Konopleva M, Garzon R, Croce C, Andreeff M (2013). CXCR4 downregulation of let-7a drives chemoresistance in acute myeloid leukemia. J Clin Invest.

[127] Zhang N, Li X, Wu CW, Dong Y, Cai M, Mok MT (2013). microRNA-7 is a novel inhibitor of YY1 contributing to colorectal tumorigenesis. Oncogene.

[128] Zhou WY, Chen JC, Jiao TT, Hui N, Qi X (2015). MicroRNA-181 targets Yin Yang 1 expression and inhibits cervical cancer progression. Mol Med Rep.

[129] Quinn JJ, Chang HY (2016). Unique features of long non-coding RNA biogenesis and function. Nat Rev Genet.

[130] Su H, Liu L, Zhang Y, Wang J, Zhao Y (2019). Long noncoding RNA NPCCAT1 promotes nasopharyngeal carcinoma progression via upregulating YY1. Biochimie.

[131] Memczak S, Jens M, Elefsinioti A, Torti F, Krueger J, Rybak A (2013). Circular RNAs are a large class of animal RNAs with regulatory potency. Nature.

[132] Hansen TB, Jensen TI, Clausen BH, Bramsen JB, Finsen B, Damgaard CK (2013). Natural RNA circles function as efficient microRNA sponges. Nature.

[133] Su M, Xiao Y, Ma J, Tang Y, Tian B, Zhang Y (2019). Circular RNAs in Cancer: emerging functions in hallmarks, stemness, resistance and roles as potential biomarkers. Mol Cancer.

[134] Conn SJ, Pillman KA, Toubia J, Conn VM, Salmanidis M, Phillips CA (2015). The RNA binding protein quaking regulates formation of circRNAs. Cell.

[135] Han J, Meng J, Chen S, Wang X, Yin S, Zhang Q (2019). YY1 Complex Promotes Quaking Expression via Super-Enhancer Binding during EMT of Hepatocellular Carcinoma. Cancer Res.

[136] Bonavida B (2017). Therapeutic YY1 Inhibitors in Cancer: ALL in ONE. Crit Rev Oncog.

[137] Hongo F, Garban H, Huerta-Yepez S, Vega M, Jazirehi AR, Mizutani Y (2005). Inhibition of the transcription factor Yin Yang 1 activity by S-nitrosation. Biochem Biophys Res Commun.

[138] Huerta-Yepez S, Vega M, Jazirehi A, Garban H, Hongo F, Cheng G (2004). Nitric oxide sensitizes prostate carcinoma cell lines to TRAIL-mediated apoptosis via inactivation of NF-kappa B and inhibition of Bcl-xl expression. Oncogene.

[139] Bonavida B, Garban H (2015). Nitric oxide-mediated sensitization of resistant tumor cells to apoptosis by chemo-immunotherapeutics. Redox Biol.

[140] Huerta-Yepez S, Baritaki S, Baay-Guzman G, Hernandez-Luna MA, Hernandez-Cueto A, Vega MI (2013). Contribution of either YY1 or BclXL-induced inhibition by the NO-donor DETANONOate in the reversal of drug resistance, both *in vitro* and *in vivo*. YY1 and BclXL are overexpressed in prostate cancer. Nitric Oxide.

[141] Sarvagalla S, Kolapalli SP, Vallabhapurapu S (2019). The Two Sides of YY1 in Cancer: A Friend and a Foe. Front Oncol.

[142] Chen Q, Yang C, Chen L, Zhang JJ, Ge WL, Yuan H (2019). YY1 targets tubulin polymerisation-promoting protein to inhibit migration, invasion and angiogenesis in pancreatic cancer via p38/MAPK and PI3K/AKT pathways. Br J Cancer.

[143] Zhang JJ, Zhu Y, Xie KL, Peng YP, Tao JQ, Tang J (2014). Yin Yang-1 suppresses invasion and metastasis of pancreatic ductal adenocarcinoma by downregulating MMP10 in a MUC4/ErbB2/p38/MEF2C-dependent mechanism. Mol Cancer.

[144] Nguyen N, Zhang X, Olashaw N, Seto E (2004). Molecular cloning and functional characterization of the transcription factor YY2. J Biol Chem.

[145] Luo C, Lu X, Stubbs L, Kim J (2006). Rapid evolution of a recently retroposed transcription factor YY2 in mammalian genomes. Genomics.

[146] Chen L, Shioda T, Coser KR, Lynch MC, Yang C, Schmidt EV (2010). Genome-wide analysis of YY2 versus YY1 target genes. Nucleic Acids Res.

[147] Klar M, Bode J (2005). Enhanceosome formation over the beta interferon promoter underlies a remote-control mechanism mediated by YY1 and YY2. Mol Cell Biol.

[148] Figiel M, Lakomska J, Milek P, Dziedzicka-Wasylewska M, Gorecki A (2019). The transcription factor YY2 has less momentous properties of an intrinsically disordered protein than its paralog YY1. FEBS Lett.

[149] Kasim V, Xie YD, Wang HM, Huang C, Yan XS, Nian WQ (2017). Transcription factor Yin Yang 2 is a novel regulator of the p53/p21 axis. Oncotarget.

[150] Kakizaki F, Sonoshita M, Miyoshi H, Itatani Y, Ito S, Kawada K (2016). Expression of metastasis suppressor gene AES driven by a Yin Yang (YY) element in a CpG island promoter and transcription factor YY2. Cancer Sci.

[151] Klar M, Fenske P, Vega FR, Dame C, Brauer AU (2015). Transcription factor Yin-Yang 2 alters neuronal outgrowth *in vitro*. Cell Tissue Res.

[152] Agarwal N, Dancik GM, Goodspeed A, Costello JC, Owens C, Duex JE (2016). GON4L Drives Cancer Growth through a YY1-Androgen Receptor-CD24 Axis. Cancer Res.

[153] Wang J, Zhou L, Li Z, Zhang T, Liu W, Liu Z (2015). YY1 suppresses FEN1 over-expression and drug resistance in breast cancer. BMC Cancer.

[154] Powe DG, Akhtar G, Habashy HO, Abdel-Fatah T, Rakha EA, Green AR (2009). Investigating AP-2 and YY1 protein expression as a cause of high HER2 gene transcription in breast cancers with discordant HER2 gene amplification. Breast Cancer Res.

[155] Wang W, Yue Z, Tian Z, Xie Y, Zhang J, She Y (2018). Expression of Yin Yang 1 in cervical cancer and its correlation with E-cadherin expression and HPV16 E6. PLoS One.

[156] Chinnappan D, Xiao D, Ratnasari A, Andry C, King TC, Weber HC (2009). Transcription factor YY1 expression in human gastrointestinal cancer cells. Int J Oncol.

[157] Luo J, Jiang X, Cao L, Dai K, Zhang S, Ge X (2014). Expression of YY1 correlates with progression and metastasis in esophageal squamous cell carcinomas. Onco Targets Ther.

[158] Kang W, Tong JH, Chan AW, Zhao J, Dong Y, Wang S (2014). Yin Yang 1 contributes to gastric carcinogenesis and its nuclear expression correlates with shorter survival in patients with early stage gastric adenocarcinoma. J Transl Med.

[159] Baritaki S, Chatzinikola AM, Vakis AF, Soulitzis N, Karabetsos DA, Neonakis I (2009). YY1 Over-expression in human brain gliomas and meningiomas correlates with TGF-beta1, IGF-1 and FGF-2 mRNA levels. Cancer Invest.

[160] Dukers DF, van Galen JC, Giroth C, Jansen P, Sewalt RG, Otte AP (2004). Unique polycomb gene expression pattern in Hodgkin's lymphoma and Hodgkin's lymphoma-derived cell lines. Am J Pathol.

[161] Erkeland SJ, Valkhof M, Heijmans-Antonissen C, Delwel R, Valk PJ, Hermans MH (2003). The gene encoding the transcriptional regulator Yin Yang 1 (YY1) is a myeloid transforming gene interfering with neutrophilic differentiation. Blood.

[162] Antonio-Andres G, Rangel-Santiago J, Tirado-Rodriguez B, Martinez-Ruiz GU, Klunder-Klunder M, Vega MI (2018). Role of Yin Yang-1 (YY1) in the transcription regulation of the multi-drug resistance (MDR1) gene. Leuk Lymphoma.

[163] Bonavida B, Kaufhold S (2015). Prognostic significance of YY1 protein expression and mRNA levels by bioinformatics analysis in human cancers: a therapeutic target. Pharmacol Ther.

[164] Kim JS, Son SH, Kim MY, Choi D, Jang IS, Paik SS (2017). Diagnostic and prognostic relevance of CP2c and YY1 expression in hepatocellular carcinoma. Oncotarget.

[165] Li M, Liu Y, Wei Y, Wu C, Meng H, Niu W (2019). Zinc-finger protein YY1 suppresses tumor growth of human nasopharyngeal carcinoma by inactivating c-Myc-mediated microRNA-141 transcription. J Biol Chem.

[166] Hafsi S, Candido S, Maestro R, Falzone L, Soua Z, Bonavida B (2016). Correlation between the overexpression of Yin Yang 1 and the expression levels of miRNAs in Burkitt's lymphoma: A computational study. Oncol Lett.

[167] Sakhinia E, Glennie C, Hoyland JA, Menasce LP, Brady G, Miller C (2007). Clinical quantitation of diagnostic and predictive gene expression levels in follicular and diffuse large B-cell lymphoma by RT-PCR gene expression profiling. Blood.

[168] Naidoo K, Clay V, Hoyland JA, Swindell R, Linton K, Illidge T (2011). YY1 expression predicts favourable outcome in follicular lymphoma. J Clin Pathol.

[169] Matsumura N, Huang Z, Baba T, Lee PS, Barnett JC, Mori S (2009). Yin yang 1 modulates taxane response in epithelial ovarian cancer. Mol Cancer Res.

[170] Jiang W, Zhao S, Shen J, Guo L, Sun Y, Zhu Y (2018). The MiR-135b-BMAL1-YY1 loop disturbs pancreatic clockwork to promote tumourigenesis and chemoresistance. Cell Death Dis.

[171] Zapata-Tarres M, Juarez-Villegas LE, Maldonado-Valenzuela A, Baay-Guzman GJ, Lopez-Perez TV, Cabrera-Munoz L (2019). Expression of YY1 in Wilms tumors with favorable histology is a risk factor for adverse outcomes. Future Oncol.

[172] de Nigris F, Botti C, de Chiara A, Rossiello R, Apice G, Fazioli F (2006). Expression of transcription factor Yin Yang 1 in human osteosarcomas. Eur J Cancer.

[173] Gashaw I, Grummer R, Klein-Hitpass L, Dushaj O, Bergmann M, Brehm R (2005). Gene signatures of testicular seminoma with emphasis on expression of ets variant gene 4. Cell Mol Life Sci.

[174] Arribas J, Castellvi J, Marcos R, Zafon C, Velazquez A (2015). Expression of YY1 in Differentiated Thyroid Cancer. Endocr Pathol.

[175] Garban HJ, Bonavida B (2001). Nitric oxide inhibits the transcription repressor Yin-Yang 1 binding activity at the silencer region of the Fas promoter: a pivotal role for nitric oxide in the up-regulation of Fas gene expression in human tumor cells. J Immunol.

[176] Xia Y, Wei K, Yang FM, Hu LQ, Pan CF, Pan XL (2019). miR-1260b, mediated by YY1, activates KIT signaling by targeting SOCS6 to regulate cell proliferation and apoptosis in NSCLC. Cell Death Dis.

[177] Lu X, Liu R, Wang M, Kumar AK, Pan F, He L (2020). MicroRNA-140 impedes DNA repair by targeting FEN1 and enhances chemotherapeutic response in breast cancer. Oncogene.

[178] Hu Z, Tie Y, Lv G, Zhu J, Fu H, Zheng X (2018). Transcriptional activation of miR-320a by ATF2, ELK1 and YY1 induces cancer cell apoptosis under ionizing radiation conditions. Int J Oncol.

[179] Ihira K, Dong P, Xiong Y, Watari H, Konno Y, Hanley SJ (2017). EZH2 inhibition suppresses endometrial cancer progression via miR-361/Twist axis. Oncotarget.

[180] Yuan P, He XH, Rong YF, Cao J, Li Y, Hu YP (2017). KRAS/NF-kappaB/YY1/miR-489 Signaling Axis Controls Pancreatic Cancer Metastasis. Cancer Res.

[181] Tang W, Zhou W, Xiang L, Wu X, Zhang P, Wang J (2019). The p300/YY1/miR-500a-5p/HDAC2 signalling axis regulates cell proliferation in human colorectal cancer. Nat Commun.

[182] Li X, Fu Q, Li H, Zhu L, Chen W, Ruan T (2019). MicroRNA-520c-3p functions as a novel tumor suppressor in lung adenocarcinoma. FEBS J.

[183] Fang Z, Yang H, Chen D, Shi X, Wang Q, Gong C (2019). YY1 promotes colorectal cancer proliferation through the miR-526b-3p/E2F1 axis. Am J Cancer Res.

[184] Liang F, Fu X, Wang L (2019). miR-5590-3p-YY1 feedback loop promotes the proliferation and migration of triple-negative breast cancer cells. J Cell Biochem.

[185] Tian JB, Cao L, Dong GL (2019). Long noncoding RNA DDX11-AS1 induced by YY1 accelerates colorectal cancer progression through targeting miR-873/CLDN7 axis. Eur Rev Med Pharmacol Sci.

[186] Qiao K, Ning S, Wan L, Wu H, Wang Q, Zhang X (2019). LINC00673 is activated by YY1 and promotes the proliferation of breast cancer cells via the miR-515-5p/MARK4/Hippo signaling pathway. J Exp Clin Cancer Res.

[187] Ye Y, Gu B, Wang Y, Shen S, Huang W (2019). YY1-Induced Upregulation of Long Noncoding RNA ARAP1-AS1 Promotes Cell Migration and Invasion in Colorectal Cancer Through the Wnt/beta-Catenin Signaling Pathway. Cancer Biother Radiopharm.

[188] Huang T, Wang G, Yang L, Peng B, Wen Y, Ding G (2017). Transcription Factor YY1 Modulates Lung Cancer Progression by Activating lncRNA-PVT1. DNA Cell Biol.

[189] Zhang JJ, Zhu Y, Zhang XF, Liu DF, Wang Y, Yang C (2017). Yin Yang-1 suppresses pancreatic ductal adenocarcinoma cell proliferation and tumor growth by regulating SOX2OT-SOX2 axis. Cancer Lett.

[190] Katsushima K, Natsume A, Ohka F, Shinjo K, Hatanaka A, Ichimura N (2016). Targeting the Notch-regulated non-coding RNA TUG1 for glioma treatment. Nat Commun.

[191] Wu Y, Zhao YM, Huan L, Zhao J, Zhou Y, Xu L (2019). A LTR retrotransposon-derived long non-coding RNA lncMER52A promotes hepatocellular carcinoma progression by binding p120-catenin.

[192] Li X, Yu M, Yang C (2020). YY1-mediated overexpression of long noncoding RNA MCM3AP-AS1 accelerates angiogenesis and progression in lung cancer by targeting miR-340-5p/KPNA4 axis. J Cell Biochem.

[193] Zhou GQ, Han F, Shi ZL, Yu L, Li XF, Yu C (2017). DNMT3A-mediated down-regulation of microRNA-105 promotes gastric cancer cell proliferation. Eur Rev Med Pharmacol Sci.

[194] Fang M, Huang W, Wu X, Gao Y, Ou J, Zhang X (2019). MiR-141-3p Suppresses Tumor Growth and Metastasis in Papillary Thyroid Cancer via Targeting Yin Yang 1. Anat Rec (Hoboken).

[195] Li J, Song J, Guo F (2019). miR-186 reverses cisplatin resistance and inhibits the formation of the glioblastoma-initiating cell phenotype by degrading Yin Yang 1 in glioblastoma. Int J Mol Med.

[196] Huang T, Wang G, Yang L, Peng B, Wen Y, Ding G (2017). MiR-186 inhibits proliferation, migration, and invasion of non-small cell lung cancer cells by downregulating Yin Yang 1. Cancer Biomark.

[197] Chen Z, Han S, Huang W, Wu J, Liu Y, Cai S (2016). MicroRNA-215 suppresses cell proliferation, migration and invasion of colon cancer by repressing Yin-Yang 1. Biochem Biophys Res Commun.

[198] Gao Y, Sun L, Wu Z, Xuan C, Zhang J, You Y (2018). miR218 inhibits the proliferation of human glioma cells through downregulation of Yin Yang 1. Mol Med Rep.

[199] Zhang Y, He S, Mei R, Kang Y, Duan J, Wei R (2018). miR29a suppresses IL13induced cell invasion by inhibiting YY1 in the AKT pathway in lung adenocarcinoma A549 cells. Oncol Rep.

[200] Chen QR, Yu LR, Tsang P, Wei JS, Song YK, Cheuk A (2011). Systematic proteome analysis identifies transcription factor YY1 as a direct target of miR-34a. J Proteome Res.

[201] Nie J, Ge X, Geng Y, Cao H, Zhu W, Jiao Y (2015). miR-34a inhibits the migration and invasion of esophageal squamous cell carcinoma by targeting Yin Yang-1. Oncol Rep.

[202] Wang AM, Huang TT, Hsu KW, Huang KH, Fang WL, Yang MH (2014). Yin Yang 1 is a target of microRNA-34 family and contributes to gastric carcinogenesis. Oncotarget.

[203] Xia B, Li H, Yang S, Liu T, Lou G (2016). MiR-381 inhibits epithelial ovarian cancer malignancy via YY1 suppression. Tumour Biol.

[204] Wang F, Li Z, Sun B (2019). miR-544 inhibits the migration and invasion of anaplastic thyroid cancer by targeting Yin Yang-1. Oncol Lett.

[205] Zhang Y, Sun Z, Zhang Y, Fu T, Liu C, Liu Y (2016). The microRNA-635 suppresses tumorigenesis in non-small cell lung cancer. Biomed Pharmacother.

[206] Jia B, Liu W, Gu J, Wang J, Lv W, Zhang W (2019). MiR-7-5p suppresses stemness and enhances temozolomide sensitivity of drug-resistant glioblastoma cells by targeting Yin Yang 1. Exp Cell Res.

[207] Fernando TR, Contreras JR, Zampini M, Rodriguez-Malave NI, Alberti MO, Anguiano J (2017). The lncRNA CASC15 regulates SOX4 expression in RUNX1-rearranged acute leukemia. Mol Cancer.

[208] Yang F, Fang E, Mei H, Chen Y, Li H, Li D (2019). Cis-Acting circ-CTNNB1 Promotes beta-Catenin Signaling and Cancer Progression via DDX3-Mediated Transactivation of YY1. Cancer Res.

[209] Zeng K, Chen X, Xu M, Liu X, Hu X, Xu T (2018). CircHIPK3 promotes colorectal cancer growth and metastasis by sponging miR-7. Cell Death Dis.

